# Quantitative phenotype analysis to identify, validate and compare rat disease models

**DOI:** 10.1093/database/baz037

**Published:** 2019-04-02

**Authors:** Yiqing Zhao, Jennifer R Smith, Shur-Jen Wang, Melinda R Dwinell, Mary Shimoyama

**Affiliations:** 1 Department of Biomedical Engineering, Marquette University and Medical College of Wisconsin, Milwaukee, WI, USA; 2 Division of Biomedical Statistics and Informatics, Mayo Clinic, Rochester, MN, USA; 3 Department of Physiology, Medical College of Wisconsin, Milwaukee, WI, USA; 4 Genomic Sciences and Precision Medicine Center, Medical College of Wisconsin, Milwaukee, WI, USA

## Abstract

The laboratory rat has been widely used as an animal model in biomedical research. There are many strains exhibiting a wide variety of phenotypes. Capturing these phenotypes in a centralized database provides researchers with an easy method for choosing the appropriate strains for their studies. Existing resources have provided some preliminary work in rat phenotype databases. However, existing resources suffer from problems such as small number of animals, lack of updating, web interface queries limitations and lack of standardized metadata. The Rat Genome Database (RGD) PhenoMiner tool has provided the first step in this effort by standardizing and integrating data from individual studies. Our work, mainly utilizing data curated in RGD, involves the following key steps: (i) we developed a meta-analysis pipeline to automatically integrate data from heterogeneous sources and to produce expected ranges (standardized phenotype ranges) for different strains and phenotypes under different experimental conditions; (ii) we created tools to visualize expected ranges for individual strains and strain groups. We developed a meta-analysis pipeline and an interactive web interface that summarizes and visualizes expected ranges produced from the meta-analysis pipeline. Automation of the pipeline allows for updates as additional data becomes available. The interactive web interface provides curators and researchers with a platform for identifying and validating expected ranges for a variety of quantitative phenotypes. The data analysis result and visualization tools will promote an understanding of rat disease models, guide researchers to choose optimal strains for their research needs and encourage data sharing from different research hubs. Such resources also help to promote research reproducibility. The interactive platforms created in this project will continue to provide a valuable resource for translational research efforts.

## Introduction

### Model organisms

Model organisms are important tools in biomedical research. Studies using model organisms have the potential to reveal the molecular mechanisms underlying disease (1–5) in human. The large-scale comparative analysis of phenotype and genotype data in model organisms can further reveal novel associations between genotypes and diseases (6–10). Such analysis traditionally has not been done as extensively in human.

*Rattus norvegicus*, or the laboratory rat, has been widely used as an animal model for physiology, immunology, neoplasia, pharmacology, toxicology, nutrition and behavior research for >160 years (11). A large number of rat strains have been bred to exhibit the phenotypes of common diseases, either spontaneously or through the application of dietary, environmental or other conditions. The rat genome sequence project completed in 2004 (12) has greatly transformed the research paradigm, creating exceptional opportunities for identifying genes and pathways contributing to disease phenotypes in rats. Results generated from rat studies can then be translated to human.

In order to leverage the power of the rat for such studies, a clear understanding of the phenotypic profiles of individual rat strains and commonly used control strains is needed. Phenotype refers to the observable morphological, physiological and behavioral characteristics of an individual under certain contexts of a study environment (13). Many phenotypic characteristics can appear or disappear, or increase or decrease, in severity throughout the lifespan of an individual. Phenotypic variation is an expression of genotype or the sum of an individual’s genetic makeup and environmental exposure. Thousands of human diseases are associated with phenotypic and genetic variations. Phenotypes observed in rats are often similar to those observed for particular human diseases, and researchers will choose particular strains as models of the disease based on these observations. However, these choices are often based on previous experiments, the researchers’ familiarity with or access to the strain or the fact that it is commonly seen by the community as a model for a particular disease. In addition, due to constraints in resources, individual investigators often focus on a limited number of phenotypes in a given strain, recording values for these few without recording a comprehensive phenotype profile of that strain.

Statistical analysis comparing phenotype values between strains is commonly done in a single experiment. However, unlike physicians in the clinic, rat researchers have not had the benefit of comprehensive expected (normal or abnormal) ranges for quantitative phenotype measurements for individual strains or for commonly used control strains based on multiple studies. The availability of statistically determined quantitative phenotype profiles for a wide range of rat strains would provide researchers with the data necessary for selecting optimal strains for their studies and help identify strains with profiles that closely mimic that of humans with particular diseases. The use of diverse panels of strains, both in phenotype and genotype, is increasing as a means to represent the diversity of human populations. Access to comprehensive quantitative phenotype profiles and comparisons with expected ranges will facilitate the assembly of such strain panels.

### Existing resources

There have been several attempts to integrate quantitative phenotype data for model organisms such as mouse and rat to provide researchers with a view of data across experiments. Current resources for rat include (i) the Rat Phenome Project of the National BioResource Project for Rat in Japan (NBRP), (ii) the PhysGen and PhysGen Knockout program and (iii) the Rat Genome Database (RGD) PhenoMiner Project. The Rat Phenome Project by NBRP in Japan, and the PhysGen program and PhysGen Knockout program include some of the most comprehensive rat phenotype measurement studies that have been conducted.

The NBRP Rat Phenome Project (http://www.anim.med.kyoto-u.ac.jp/nbr/phenome.aspx) (14) is a good reference to rat physiology given that the studies construct measurement groups with the same number of animals (six rats) and on both sexes. Researchers can compare the same phenotype across strains, and between-study variance can be controlled by using the same number of animals. In addition, conclusions about sex differences in phenotypes will be easier to draw since the measurements were done under the same conditions and at the same age.

However, there are some drawbacks to NBRP Rat Phenome Project: (i) the number of animals (six rats) they used was small, resulting in relatively large within study variance; (ii) the measurement method used by NBRP may not be available in other laboratories. As a result, NBRP phenotype measurements may not be representative and make it hard for researchers to compare their own results with NBRP measurements; (iii) their project is a one-time effort for each strain. However, even inbred rat strains can drift in their genetic make-up or physiological characteristics due to environmental influence. This again makes it hard for researchers to compare their own results with NBRP measurements. Thus, it is essential to continuously measure rat phenotypes and then compare or combine old measurement values with new ones.

The PhysGen Program for Genomic Application at the Medical College of Wisconsin has produced large-scale phenotype data using a variety of inbred and consomic rat strains. Comprehensive characterization (434 845 physiological data points) of these consomic strains, each carrying a chromosome from the sequenced Brown Norway strain, allowed for immediate mapping of traits to a particular chromosome without the need for genetic crosses (15). The addition of mutant strains in the PhysGen Knockout program allowed *in vivo* studies to be done to study the role of genes in pulmonary, cardiovascular, vascular and renal diseases. The advantage of The PhysGen program is that it created a federated database with curated measurements on rats from different laboratories and studies on different rat strains (inbred, mutant and consomic). The PhysGen program developed web tools that enable querying of experiments for a specific phenotype. The PhysGen website provided visualization of individual phenotype results across multiple strains with statistical analysis. It also provides strain profiles that summarize both general and phenotype data for individual strains (16).

However, there are several drawbacks to PhysGen data presentation: (i) the web interface only enables queries within a certain protocol or experiment, e.g. BIOCHEMISTRY, CARDIAC, RENAL and RESPIRATORY. However, some phenotypes were measured in multiple protocols. For example, because individuals with a high resting heart rate and a low beat-to-beat heart rate variability have an increased risk of developing kidney disease (17), `heart rate’ was measured in both CARDIAC and RENAL, which makes phenotype-based comparison and integration difficult; (ii) it lacks a visible data standardization process so that the measurement method, experimental condition and age of rats used remain unclear behind those data points. A user needs to refer to the protocol to gain this information. This again makes it difficult to truly compare data from various sources and across experiments.

The RGD (rgd.mcw.edu) is the most comprehensive data repository and informatics platform for the laboratory rat (18). RGD curates and integrates data from published literature, individual research projects as well as the PhysGen Program for Genomic Application (19) and the NBRP Rat Phenome Project in Japan (14). Data include information on strains, Quantitative Trait Loci (QTLs) and experimental phenotype measurements across hundreds of strains. To better curate and query comprehensive experimental data from heterogeneous data sources, RGD initiated the PhenoMiner project (https://rgd.mcw.edu/rgdweb/phenominer/home.jsp) (20). Video tutorials about how to effectively use this database are described on RGD website (https://rgd.mcw.edu/wg/home/rgd_rat_community_videos/rgd-tool-and-website-videos/), and updates on new tools were described in the accompanying paper by Wang *et al.* (20) in this issue.

The advantages of PhenoMiner are (i) it standardizes quantitative phenotype records for rat strains, clinical measurements, measurement methods and experimental conditions using ontologies [rat strain ontology (RSO) (21), clinical measurement ontology, measurement method ontology and experimental condition ontology (22, 23), respectively]; (ii) this standardization allowed for the integration of data from large scale and small scale phenotype projects; (iii) users can query and retrieve data from multiple experiments and visualize results; and (iv) users can also download retrieved data.

While systematic data integration and visualization in PhenoMiner enabled comparisons on quantitative phenotypes across experiments, the drawback of the current PhenoMiner portal is its limited ability for statistical integration of data. Further quantitative analysis using a standardized statistical tool would provide more insights in understanding rat strains in terms of disease models. Moreover, an integrated analysis of overall strain phenotype measurement is desirable for researchers to better understand cross strain differences. Currently researchers often choose strains for use as disease models using data points from a limited number of experiments or based on availability, prior use or familiarity. The availability of statistically determined expected ranges for quantitative phenotypes for multiple individual strains, for those often used as controls and for rat in general would improve the ability of investigators to choose appropriate strains for their studies objective and assist them in examining potential factors that might cause measurement variation. The expected range would also provide a standard interpretation of experimental results from different laboratories.

### Motivation and aims

Therefore, the motivation for our work is to take advantage of the substantial volume of quantitative phenotype data in the RGD to establish expected ranges for different rat strains. To achieve this goal, our work involved the following key steps: (i) establish a standardized phenotype range for different rat strains using the meta-analysis method and (ii) create tools to mine and visualize data for individual strains and across strains. In the first step, we conducted a meta-analysis to effectively synthesize archived phenotype data in the PhenoMiner database; stratify each population based on strain (inbred/outbred/congenic/transgenic/mutant), gender, age, etc.; and produce comparable expected ranges of important physiological phenotypes (such as heart weight and systolic blood pressure). Statistical tests will also be performed to assess differences between different strains of certain phenotypes. The result from this work will greatly benefit researchers using rat models in determining a proper strain, age, gender and all relevant parameters for their studies.

## Materials and methods

Meta-analysis is a powerful tool for determining expected ranges for particular phenotype measurements across multiple experiments. This approach involves statistical techniques for combining measurements or findings from independent studies to draw insights on a specific research question. It is often used to assess the effectiveness of clinical treatments by combining data from several randomized control trials. It provides a precise estimate of treatment effect, overcoming biases that could occur when examining a single study and it offers a systematic synthesis of the experimental data. A recent research study revealed that single-laboratory studies with large sample size produce results that are more precise but less accurate and therefore less reproducible (24). By contrast, multi-laboratory designs including as few as two to four laboratories increased coverage probability by up to 42 percentage points without a need for larger sample sizes. They also demonstrated that within-study standardization is a major cause of poor reproducibility between studies (24).

A systematic review methodology is essential as the first step of meta-analysis. The objective of a systematic review is to present a balanced and impartial summary of the existing research, enabling synthesis of all relevant studies of adequate quality (25). This stresses the need to take great effort and care to find all the relevant studies (published and unpublished) and to assess the methodological quality of the design and execution of each study (26). The standardized and integrated data at RGD is a good resource of systematically managed experimental phenotype measurements. It includes both data from published studies from current biomedical literature as well as large-scale data from rat community repositories such as PhysGen (19) and the Rat Phenome Project (14).

**Figure 1:**
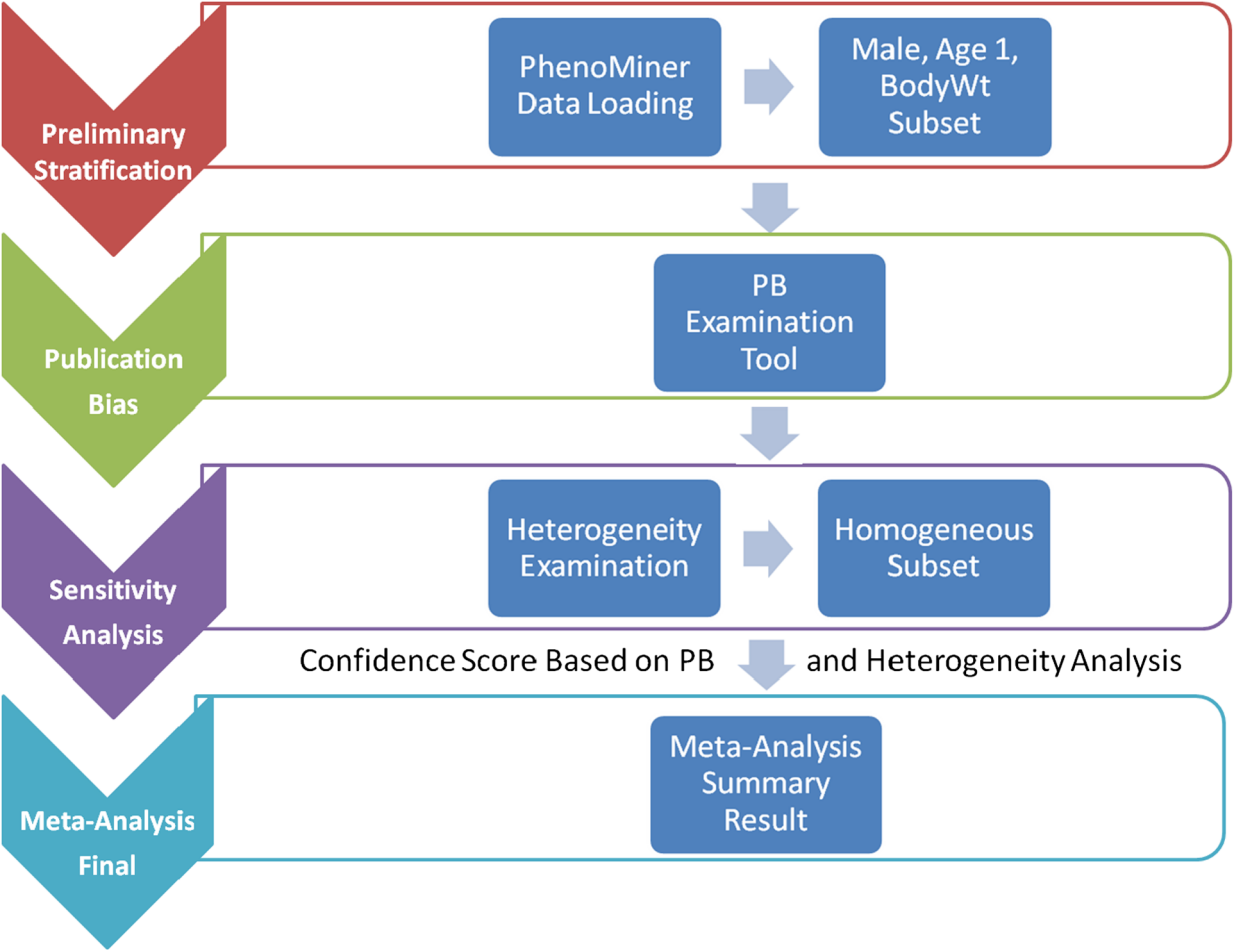
System pipeline for meta-analysis. (1) Preliminary stratification: Choose a subset of phenotype measurements based on preliminary stratification, which included strain (including similar strains inbred at different locations and substrains), sex, age group and phenotype measurement methods. (2) Publication bias: One key concern is publication bias, which arises because experiments with negative findings are less likely to be published than those that highlight results which support hypotheses. We used funnel plot to examine any publication bias. (3) Sensitivity analysis: Data with poor quality for non-systematic reasons are often an issue in meta-analysis so selection, inclusion and integration (or population stratification) of data are an important factors for consideration, which can be completed through sensitivity analysis. (4) Meta-analysis result summary: Results will be displayed in a forest plot. The *x*-axis is the value of measurement or effect size. Each datum is shown as a blob or square. The size of the blob or square is proportional to the sample size. A horizontal line representing 95% confidence interval is drawn through the center of each study’s square to represent the uncertainty of the measurement.

Meta-analysis is not just a single statistical analysis; it involves a pipeline of preliminary stratification, exploratory decision-making (publication bias and sensitivity analysis) before the final statistical meta-analysis can be performed. The pipeline for analyzing RGD PhenoMiner data consists of four major components (Figure 1). In the following sections, we will introduce the methods for each step in the pipeline followed by its corresponding results since results from each step are useful in deciding the method used in the next step. In addition to developing the algorithms for each component, a user interface was created to facilitate determination of appropriate parameters and to dynamically implement the workflow needed for the analyses (described in further detail below).

### Preliminary stratification

**Figure 2:**
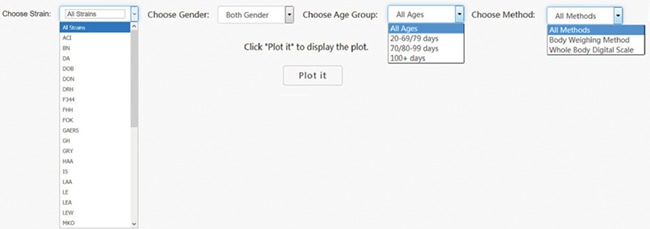
PhenoMiner preliminary stratification for body weight. Interface was created to dynamically conduct the preliminary stratification step. Options for strain and phenotype measurement methods depend on the major phenotype under analysis. Age group divisions were decided by expert heuristic definitions of young, adult and old rats. Age group division can be different. For example, for body weight, 20–79 and 80–99 days might be a good way to divide young and adult rats, but for other cardiovascular phenotypes, 20–69 and 70–99 days are a good way to divide young and adult rats.

First it was necessary to choose a subset of phenotype measurements based on preliminary stratification, which included strain (including similar strains inbred at different locations and substrains), sex, age group and phenotype measurement methods. Figure 2 shows the interface created to dynamically conduct the preliminary stratification step. Options for strain and phenotype measurement methods depend on the major phenotype under analysis. Age group divisions were decided by expert heuristic definitions of young, adult and old rats. However, for different phenotypes, young and adult rat age group can have very different phenotype measurement values that always lead to high heterogeneity and affects meta-analysis quality. As a result, age group division is a data-driven heuristic score with expert input provided prior definition.

### Publication bias with funnel plot

Because much of the data included in this study arise from published research, one key concern is publication bias, which arises because experiments with negative findings are less likely to be published than those that highlight results that support hypotheses (27). Funnel plots can be used to assess the presence of publication bias (28) by displaying the studies included in the meta-analysis in a plot of measurement value or effect size (explained in detail in the statistical analysis section) against sample size or another measure of precision (29, 30). The expected picture should be a symmetrical inverted funnel (31). This is in accordance with the assumption that smaller studies have more chance of variability than larger studies. Figures 1 and 2 in (32) are examples of symmetrical and asymmetrical funnels. An asymmetric plot suggests (i) smaller studies showing no effect might be missing or (ii) small studies tend to have larger effect sizes (33). The first reveals a true publication bias while the second does not. There are also controversies over the use of funnel plots due to disputes over appropriate interpretation of asymmetry (34–36). For example, true heterogeneity in study population (due to subgroups with a different intervention effect) will lead to funnel plot asymmetry (36). In addition, chance is also critical for interpretation of funnel plot asymmetry since most meta-analyses in the biomedical field contain few studies (37). Therefore, we need to examine closely before reaching a conclusion of publication bias (38).

In 1997, Egger *et al.* (28) proposed an estimator for visualizing asymmetry in the funnel plot. In addition to the simple visualization of asymmetry, they also used a regression test to measuring asymmetry quantitatively. The regression test is a linear regression of normalized effect size estimate (value/SD) against precision (1/SD). The assumption of the regression test is that a homogeneous set of trials (without publication bias) will regress toward a line that runs through the origin (intercept,0), with the slope indicating the size and direction of effect (39). When the regression line runs through the origin, it indicates a symmetrical funnel plot. However, the Egger test has a relatively high false positive rate (higher type I error rate).

**Figure 3:**
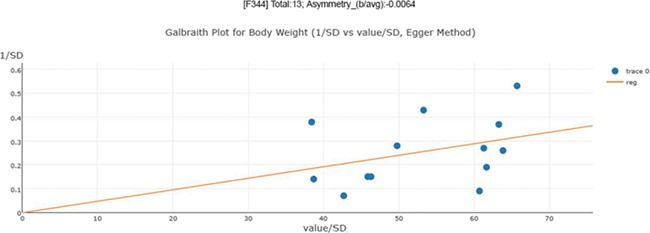
Publication bias examination demonstrations for body weight. The regression test is a linear regression of normalized effect size estimate (value/SD) against precision(1/SD). When the regression line runs through the origin, it indicates a symmetrical funnel plot. An asymmetry score is calculated as the ratio of intercept for the regression line to average value for the measurements in the group under analysis.

An asymmetry score is calculated as the ratio of intercept for the regression line to average value for the measurements in the group under analysis (Figure 3): $\mathrm{Asy}=\frac{\mathrm{intercept}}{\mathrm{average}\_\mathrm{value}}$ .

### Optimal number of experiments for meta-analysis quality control

**Figure 4:**
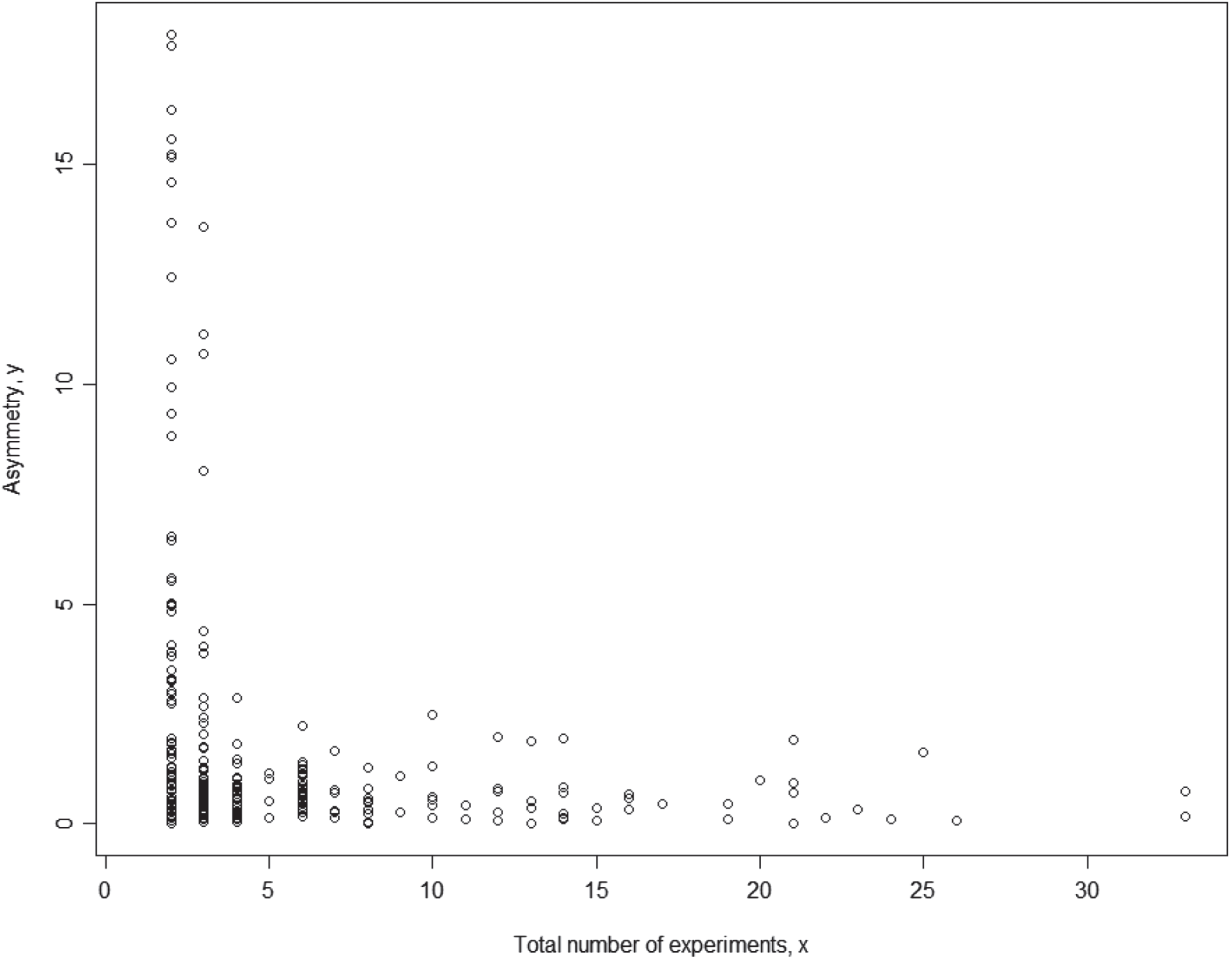
Relationship between asymmetry score and total number of experiments for body weight. While each meta-analysis is based on a unique phenotype-strain pair, for different phenotype-strain pairs, the total number of experiments can vary significantly. The result above shows that the asymmetry score (Asy) was reduced significantly with ***four*** or more experiments in one meta-analysis.

To assure the quality of meta-analysis, we need to assign a confidence level to our meta-analysis model (here we used a binary parameter with value `confident’ and `low_confidence’) and results given a specific set of phenotype measurement data (in a single meta-analysis). While each meta-analysis is based on a unique phenotype–strain pair, for different phenotype–strain pairs, the total number of experiments can vary significantly. Hence, we examined the relationship between asymmetry score (Asy) from publication bias analysis and total number of experiments (in a single meta-analysis) to identify potential biases in our analysis. The example below used body weight data (Figure 4). The result shows that the asymmetry score (Asy) was reduced significantly with ***four*** or more experiments in one meta-analysis.

We also examined range distribution and its relationship with the total number of experiments (in a single meta-analysis). Figure 4 shows that data range for different meta-analysis datasets also varies with total number of experiments for each meta-analysis. The range is defined as the difference between maximum value among all studies and minimum value among all studies. For a meta-analysis with fewer experiments, the range between studies can be falsely small. This indicates that meta-analysis without enough experiments might have false negative heterogeneity representations (heterogeneity not revealed) and thus the meta-analysis model choice might be wrong (fixed-effect or random-effect).

### Sensitivity analyses with forest plot

Data with poor quality for non-systematic reasons is often an issue in meta-analysis so selection, inclusion and integration (or population stratification) of data are an important factors for consideration. Those decisions may affect major findings, so researchers usually carry out some sensitivity analysis prior to integration of data. The usual way of displaying data for sensitivity analysis is by a forest plot. This displays the findings from each individual study using a blob or square (40); the *x*-axis is the value of measurement or effect size. The size of the blob or square is proportional to the sample size. A horizontal line representing 95% confidence interval is drawn through the center of each study’s square to represent the uncertainty of the measurement. The meta-analysis result is displayed as a diamond.

After exploring the forest plot of the study cohort, the main findings can be changed by varying the approach to integration (or population stratification). An effective sensitivity analysis will explore the effect of excluding various categories of studies, such as outlier data (outliers need to be excluded for justifiable reasons), data without specified sex information or data from unpublished studies. It may also examine how consistent the results are across various subgroups (perhaps defined by subject population stratification, type of measurement method or condition).

A useful sensitivity analysis is a series of repeated meta-analyses, usually omitting one study at a time. A heterogeneity score is calculated and the meta-analysis model (fixed-effect or random-effect) is chosen based on the heterogeneity score. Such an ‘exclusion sensitivity plot’ by Bax *et al.* (41) reveals any study/observation that has a particularly large influence (outlier) on the result of the meta-analysis. An interactive interface in our tools provides users with the ability to decide on inclusion/exclusion of any study/observation in the meta-analysis before proceeding with the next step in the analysis. Additionally, users of the pipeline may identify subgroups in their data and may decide to adopt a modified sensitivity analysis by excluding a group of studies/observations and creating new stratification criteria. For example, users may find substrains A/X and A/Y to have different measurements for phenotype T. Instead of analyzing phenotype T using all data for strain A, users may want to further stratify the population using substrain characteristics. After initial analysis, we found that the large value of the outlier is due to sample age. Sixty-five days might not be a homogenous group member for young rat group in terms of body weight although it might be acceptable for another phenotype.

### Statistical meta-analysis

#### Cochrane’s Q for heterogeneity

In the meta-analysis, we needed to evaluate whether the results from different studies can be `combinable’. This involved examining heterogeneity. Statistics commonly used for testing heterogeneity include Cochrane’s Q, a statistic based on the χ^2^ test and the *I*^2^ statistic. Cochrane’s Q test aims to offer a standardized heterogeneity comparison among different measurements, similar to the idea behind the *t*-test. But it was unsatisfactory as it depends heavily on the scale of measurement and has no absolute interpretation for comparison. Thus the *I*^2^ statistic was more attractive because it scores heterogeneity between 0% and 100%, with 25% corresponding to low heterogeneity, 50% to moderate and 75% to high (42). It interprets the percent of the total variance that is due to between-study heterogeneity. However, both methods may sometimes fail to detect heterogeneity when it is actually present (43). In addition, meta-analysis parameters are very data dependent; we proposed a visual method to find the optimized cut-off threshold for our specific dataset.

If the study results for a sub-population are relatively homogenous, we can integrate the results using a general meta-analysis method (fixed-effect model). If heterogeneity exists, we can further stratify the current sub-population based on a conceptual stratification method (e.g. stratify based on age and sex) or use random-effect model meta-analysis. If the heterogeneity was caused purely from systematic variations between studies, then we would need to define special statistical parameters (inter-study variance used in random-effect model) to interpret the systematic heterogeneity of the results.

#### *I* ^2^ statistics cut-off threshold

**Figure 5:**
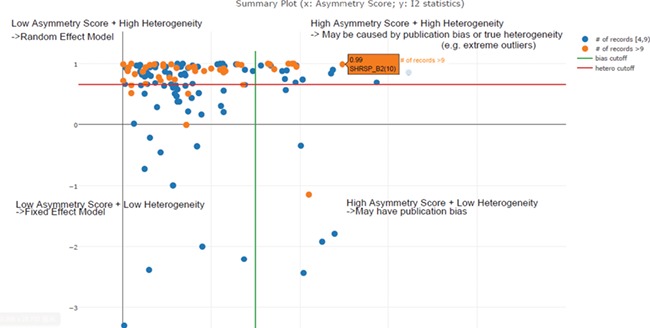
Scatter plot of asymmetry score and I2 statistics for different meta-analysis results for body weight data (*x*: asymmetry score; *y*: I2 statistics). The four quadrants represent different characteristics of datasets for each meta-analysis task. Quadrant 1 (top right) represents a high asymmetry score and high heterogeneity, which may be caused by publication bias or true heterogeneity (e.g. extreme outliers). Quadrant 2 represents a high asymmetry score and low heterogeneity, which may indicate true publication bias. Quadrant 3 represents a low asymmetry score and low heterogeneity, which indicates that we should choose the fixed-effect model. Quadrant 4 represents a low asymmetry score and high heterogeneity, which indicates that we should choose the random-effect model. From this summary plot, we can determine that *I^2^* ***= 0. 85*** (represented by the red line) is an optimal cut-off threshold to separate high and low heterogeneity datasets.

In the previous step, we decided to exclude or take a lower confidence in meta-analysis when the total number of experiments was below four. In this step, we needed to decide the cut-off threshold for the *I*^2^ statistic to decide the model choice for each meta-analysis. In the example in Figure 5, we can see that *I*^2^ **=** 0.85 (represented by the red line) is an optimal cut-off threshold to separate high- and low-heterogeneity datasets. The four quadrants in Figure 5 represent different characteristics of datasets for each meta-analysis task. Quadrant 1 (top right) represents a high asymmetry score and high heterogeneity, which may be caused by publication bias or true heterogeneity (e.g. extreme outliers). Quadrant 2 represents a high asymmetry score and low heterogeneity, which may indicate true publication bias. Quadrant 3 represents a low asymmetry score and low heterogeneity, which indicates that we should choose the fixed-effect model. Quadrant 4 represents a low asymmetry score and high heterogeneity, which indicates that we should choose the random-effect model. From this summary plot, we can determine model choice for data in Quadrants 3 and 4. For data in Quadrants 1 and 2, we need to further confirm the existence of publication bias before any conclusions can be made.

#### Fixed-effect model versus random-effect model

The presence or absence of heterogeneity influences the subsequent method of analysis. If heterogeneity is absent, then the analysis employs a fixed-effect model. This assumes the size of the system effect is fixed across all studies and the variation seen between studies is completely random. However, as studies generally vary in size and variance, each study is considered to have different precision. In meta-analysis, the key concept is to assign a weight to each study while synthesizing results. Generally believed by statisticians, a study based on 100 subjects is assumed to provide a more `precise’ estimate than a study based on 10 subjects. Therefore, larger studies should carry more `weight’ in the analyses than smaller studies. This sample size-based approach is a simple one. A better approach is to assign weight by the inverse variance (${w}_i=\frac{1}{{{\mathrm{s}}_{\mathrm{i}}}^2}$). Thus, the meta-analysis mean is ${\mathrm{m}}_{\mathrm{w}}$ = $\sum_{\mathrm{i}}{\mathrm{w}}_{\mathrm{i}}{\mathrm{y}}_{\mathrm{i}}\!\Big/ \!\sum_{\mathrm{i}}{\mathrm{w}}_{\mathrm{i}}$ and variance $\operatorname{var}\Big({\mathrm{m}}_{\mathrm{w}}\Big)=1\!\Big/ \!\sqrt{\sum_{\mathrm{i}}{\mathrm{w}}_{\mathrm{i}}}$.

Ideally, population stratification should be sufficient for removing heterogeneity, and we should be able to construct our meta-analysis model with the fixed-effect model as mentioned above with inverse variance.

Another more commonly used way is to adopt a random-effect model. Random-effect models assume that the treatment effect varies between studies. The observed measurement ${\mathrm{y}}_{\mathrm{i}}$, from the *i*-th study is made up of two additive components: the true measurement, ${\theta}_{\mathrm{i}}$, and the sampling error, ${\mathrm{e}}_{\mathrm{i}}$. That is, ${\mathrm{y}}_{\mathrm{i}}$=${\theta}_{\mathrm{i}}$+${\mathrm{e}}_{\mathrm{i}}$ for i = 1, …, k. The variance of ${\mathrm{e}}_{\mathrm{i}}$, can be estimated by ${{\mathrm{s}}_{\mathrm{i}}}^2$. Additionally, inter-study variance has to be considered in the formula. The first and most widely adopted random-effect models for meta-analysis was proposed by DerSimonian and Laird in 1986 (44). This method is now considered the `standard approach’ for meta-analysis in medical and clinical research. In their model, they also adopted inverse variance weight. The total variance, however, is the sum of within-study variance (${{\mathrm{s}}_{\mathrm{i}}}^2$) and inter-study variance (${\tau}^2$), leading to weight as ${w}_{\mathrm{i}}=\frac{1}{\tau^2+{{\mathrm{s}}_{\mathrm{i}}}^2}$. ${\tau}^2$ used in our analysis was derived from DerSimonian and Laird’s (44) non-iterative estimator.

### Meta-analysis workflow

**Figure 6:**
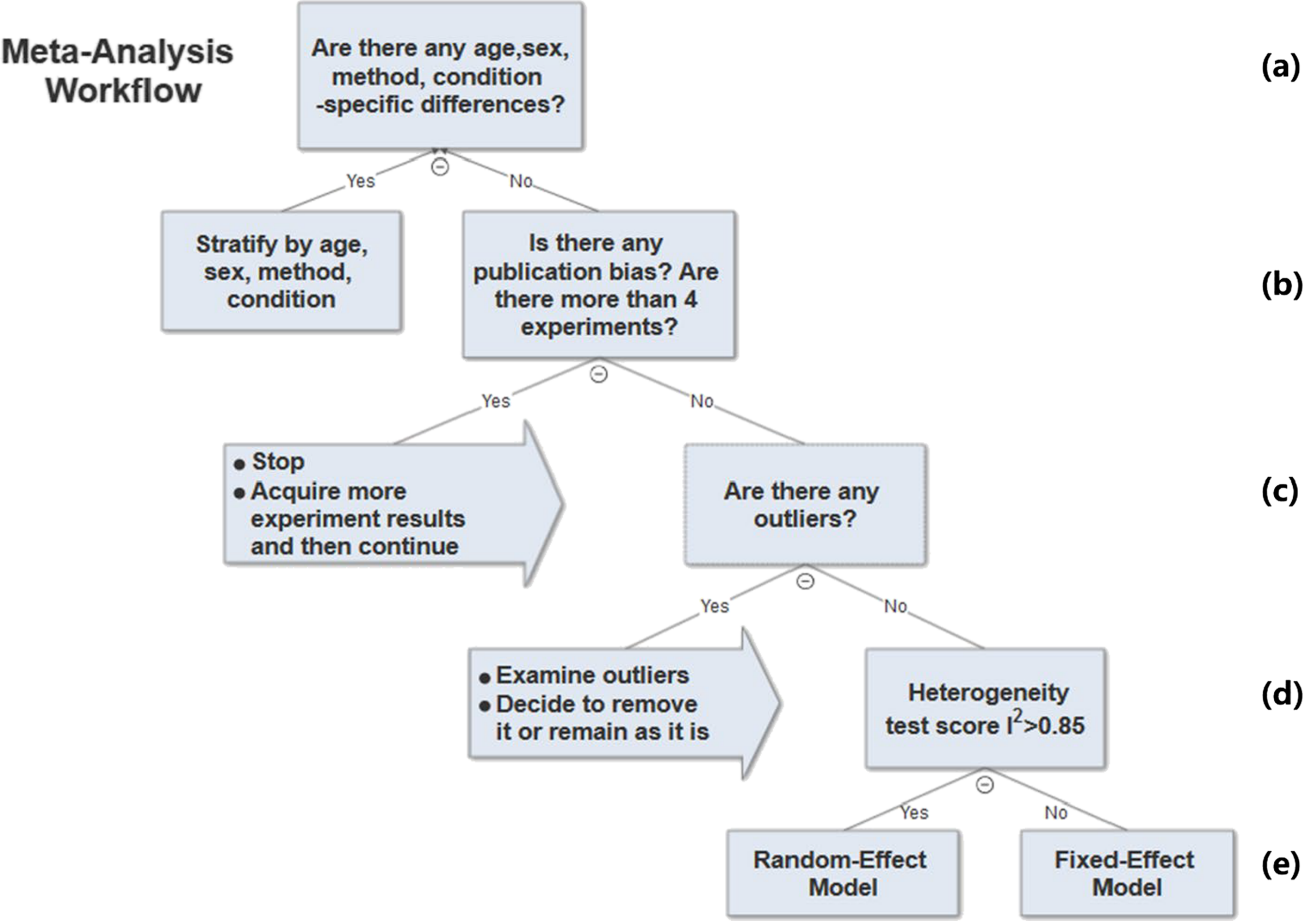
Meta-analysis workflow. In summary, our workflow is composed of the following key steps: (a) Preliminary stratification by age, sex, method and experimental condition. (b) Perform exploratory analysis to examine publication bias and total number of experiments. (c) Perform exploratory analysis to determine inclusion/exclusion of individual study/observation in the meta-analysis. (d) Examination of heterogeneity using Cochrane’s Q, a statistic based on the χ^2^ test and the *I*^2^ statistic. (e) The fixed-effect and random-effect model choice threshold is set to *I*^2^ = 0.85, which is considered the optimal threshold to distinguish heterogeneity caused by a limited number of records or true inter-study variance. (f) All the summary values for the phenotype under analysis are displayed in a summary forest plot.

The previous sections described the analysis methods we used to determine parameters in the meta-analysis workflow. A decision tree of the workflow is shown in Figure 6.

**Figure 7:**
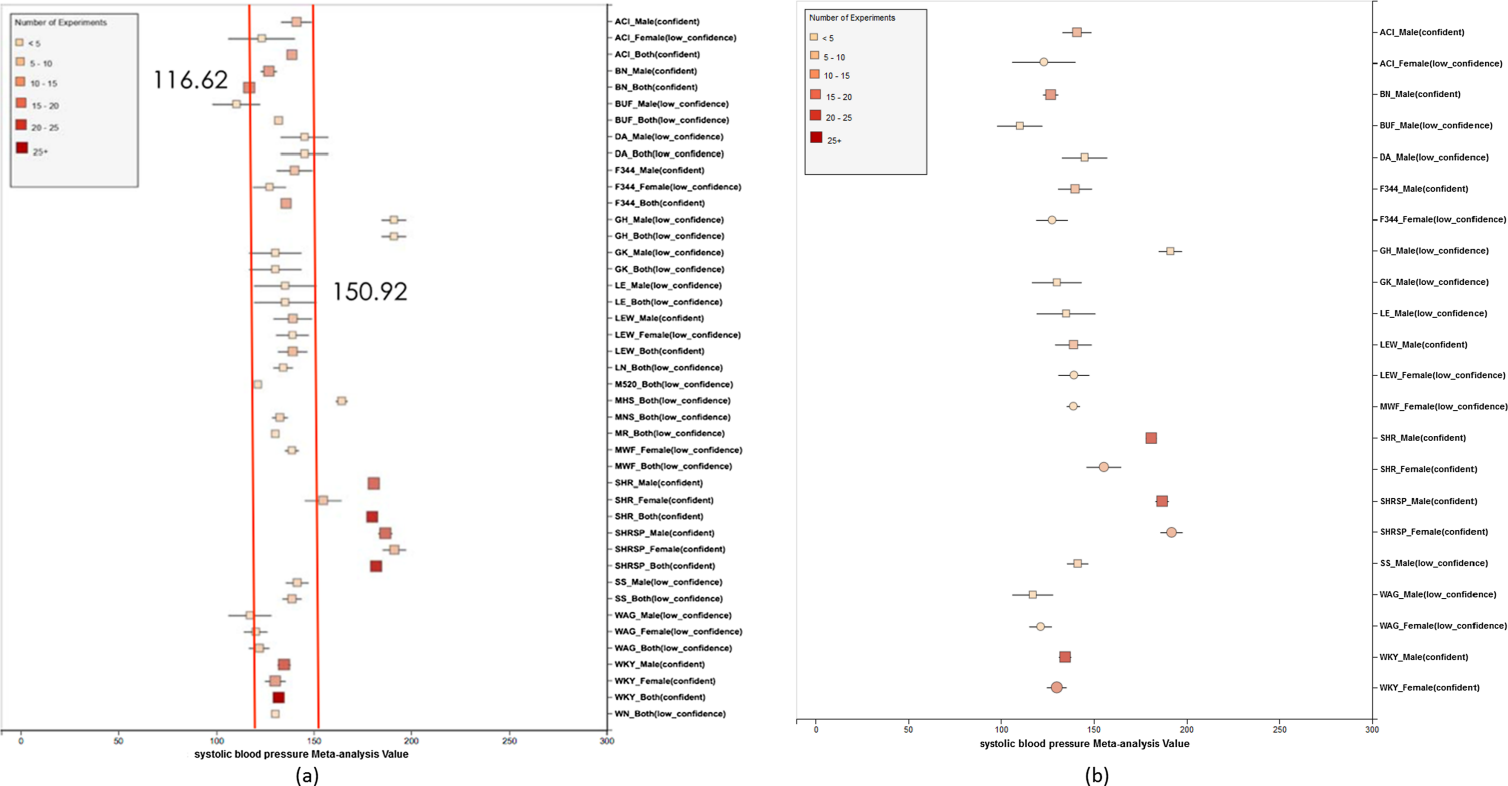
(a) Forest plot of meta-analysis summary for `Systolic Blood Pressure’. The center of the box represents the meta-analysis mean and the range determined by one standard deviation above and below the meta-analysis value. The color of the boxes showed the total number of experiments that made up of the meta-analysis range. It is a sign of confidence for the resulting phenotype range. On the right side, the legend shows the strain and sex of which the range is representing. In the bracket, we also noted the confidence level of analysis considering the total number of experiments (<4, low_confidence; ≥, 4 confident). (b) Forest plot with different indicator shapes for different genders. Users will have the option to choose display between (a) and (b).

In summary, our workflow is composed of the following key steps:

i. Perform preliminary stratification by age, sex, method and experimental condition (Figure 6a).
ii. Perform exploratory analysis (Figure 6b) to examine publication bias and total number of experiments. For examination of publication bias, we used the original Egger test considering the trade-off between power and type I error rates. |Asymmetry score| > 1.5 or total number of experiments <4 is a sign of a potentially biased sample. Thus, conclusions from the meta-analysis might not be trustworthy. We will need to acquire more data in order to proceed with the analysis.
iii. Perform exploratory analysis (Figure 6c) to determine inclusion/exclusion of individual study/observation in the meta-analysis.
iv. Examination of heterogeneity (Figure 6d) using Cochrane’s Q, a statistic based on the χ^2^ test and the *I*^2^ statistic. For each set of experiments qualified for meta-analysis, we calculated Q and *I*^2^, which were used to determine model selection in the next step.
v. Choosing meta-analysis model (fixed effect or random effect). The fixed-effect and random-effect model choice threshold is set to *I*^2^ = 0.85, which is considered the optimal threshold to distinguish heterogeneity caused by a limited number of records or true inter-study variance.
vi. All the summary values for the phenotype under analysis are displayed in a summary forest plot (for example, see Figure 7). The center of the box represents the meta-analysis mean and the range determined by one standard deviation above and below the meta-analysis value. The color of the boxes showed the total number of experiments that made up of the meta-analysis range. It is a sign of confidence for the resulting phenotype range. On the right side, the legend shows the strain and sex of which the range is representing. In the bracket, we also noted the confidence level of analysis considering the total number of experiments (<4, low_confidence; ≥4, confident).

## Results

The analysis pipeline serves as a curation tool, thus was not publicly available. The Expected Ranges tool (https://rgd.mcw.edu/rgdweb/phenominer/phenominerExpectedRanges/views/home.html) can be accessed from the Phenotypes and Models icon on the RGD homepage. We decided to focus on a use case in cardiovascular area because rat is mostly used in cardiovascular research. Our meta-analysis provided expected ranges for 24 cardiovascular-related phenotypes. We analyzed all the available strains for which whole genome sequence is available for each phenotype. In terms of non-sequenced strains, we focused on MWF since most non-sequenced strains had limited data available for these phenotype areas.

In the first step, our meta-analysis used only experiment records under control conditions for inbred stains. In this way, the meta-analysis range can be regarded as an `Expected Range’ for a specific strain or strain group. One type of strain group was created by grouping all substrains under a certain parent strain name according to the RSO. In accordance with official standards, a strain is given a substrain designation when it is bred for 20 generations or more in a different facility or laboratory. For example, strain group `ACI’ includes the substrains `ACI/Eur’, `ACI/Kun’, `ACI/N’, `ACI/SegHsd’, `ACI/Ti’ and `ACI/Ztm’. Control strain groups were also created based on their widespread use as control animals and acceptance as exhibiting `normal measurements. For example, the `Normal Systolic Blood Pressure Strain Group’ consists of strains that are considered commonly used as controls in blood pressure experiments and to exhibit `normal systolic blood pressure’. The `Normal Systolic Blood Pressure Strain Group’ was created in an iterative process using a domain expert with extensive experience in large scale phenotyping projects: (i) Strains commonly used as controls were identified based on experience and prior knowledge and designated as `founder control strains’, e.g. BN and WKY that have long been used as control models. (ii) An initial `Expected Range’ was constructed based on phenotype ranges of those `founder’ strains using the highest and lowest values of the previously determined expected ranges for each strain. (iii) The overlap of previously determined expected ranges of other strains with this initial `Control Expected Range’ was examined to determine whether additional strains could be included in the normal phenotype strain group. (iv) An updated `Control Expected Range’ was constructed using all strains added to the normal phenotype strain group.

In addition to constructing a general `Expected Range’, we stratified our analysis by age, sex and measurement method when data were available for different ages, sexes or methods. We then constructed age-, sex- and method-specific `Expected Ranges’ using the same workflow. The results of constructing phenotype `Expected Ranges’ are discussed in the first section below.

After the initial `Expected Ranges’ for 24 phenotypes using inbred strains were constructed, we also performed meta-analysis for outbred and mutant strains, when sufficient data existed. We also evaluated the applicability of the analysis under non-control conditions using inbred strains in which a measured salt diet was the experimental condition. Creating such expected ranges for particular experimental conditions will further assist researchers in choosing model strains for particular experiments and provide data for developing tools and statistical processes that would allow them to analyze their own data.

Phenotype data were available for inbred, outbred, consomic, congenic, mutant and transgenic strains. However, for this study, initial development of the algorithms and workflows and expected ranges of phenotypes were established using only inbred strains. In addition, phenotype records in which experimental conditions equivalent to `naïve control’ were used whereas those involving experimental diets, exercise, application of drugs or chemicals or other manipulated conditions were not initially used in this study. Figure 7 shows an example of a forest plot summary produced for `systolic blood pressure’ for different age groups. We were then able to identify strains with expected ranges within or overlapping the previously constructed `Control Expected Range’ (ACI, BN, BUF, DA, F344, GK, LE, LEW, LN, M520, MNS, MR, MWF, and WKY) and strains with expected ranges outside of the constructed `Control Expected Range’ (GH, LH, MHS, SHR, and SHRSP) from the graph. Figure 9 shows phenotype expected ranges for `systolic blood pressure’ for outbred and mutant strains under naïve control conditions.

**Figure 8:**
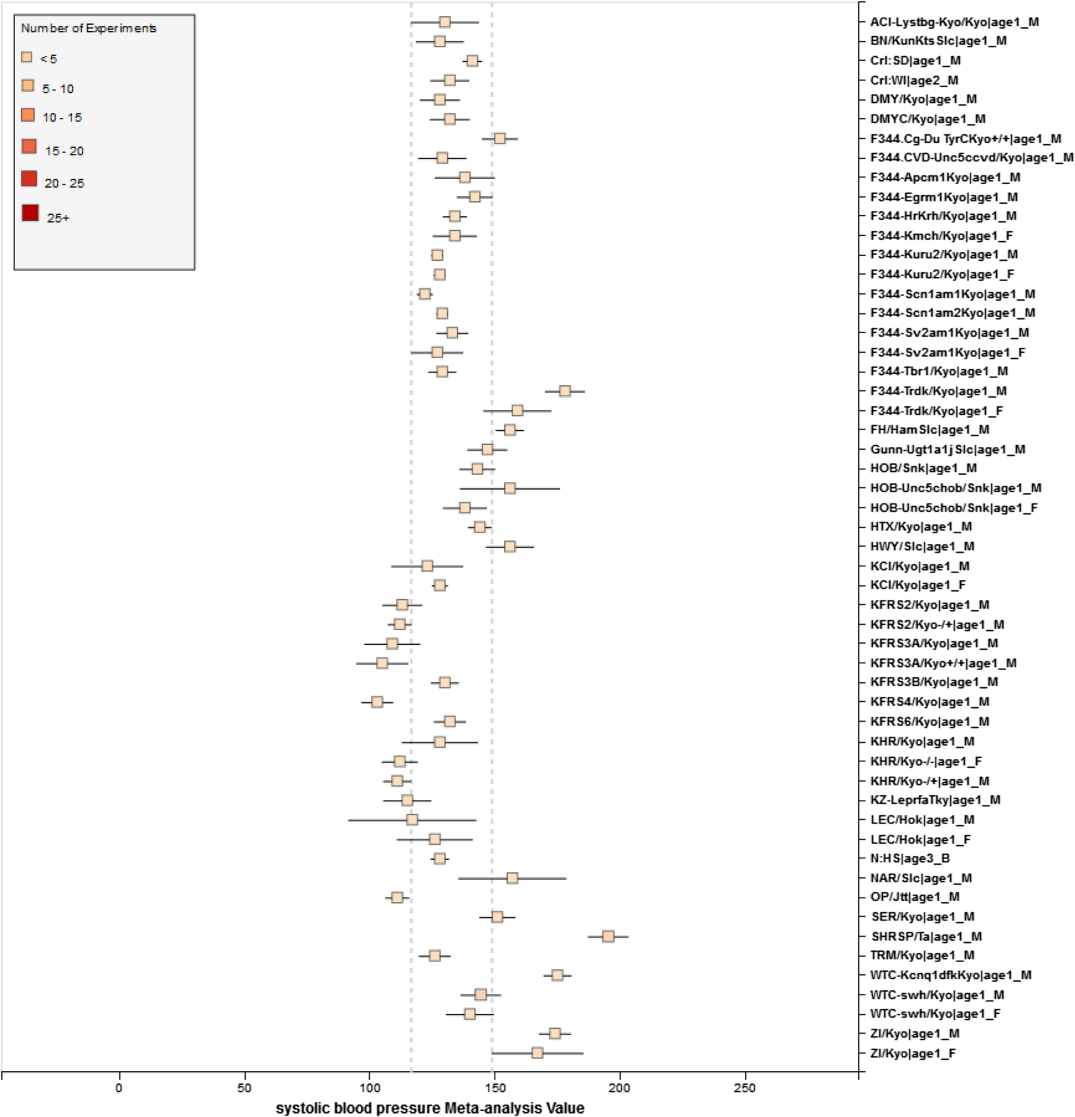
RGD phenotype expected ranges for `Systolic Blood Pressure’ for outbred and mutant strains under naïve control conditions. The dotted lines represent the expected range for the `Control Strain Group’ based on 14 inbred strains used as controls for this phenotype.

**Figure 9:**
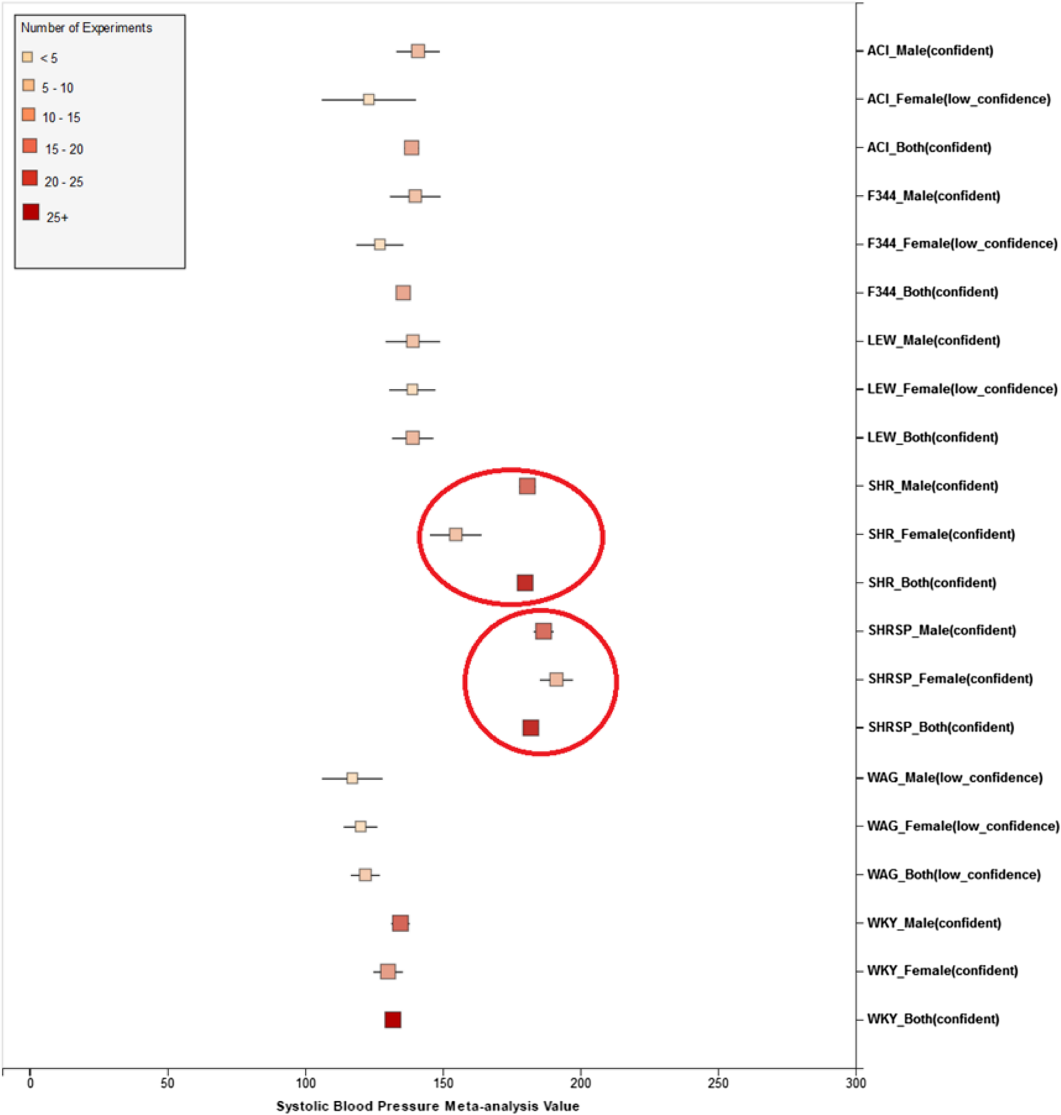
Summary for `Systolic Blood Pressure’ shows sex pattern does not always hold true: the sex specific pattern for SHRSP is opposite of the common pattern as female SHRSP rates have higher blood pressure. On the other hand, SHR exhibits the common pattern.

Supplementary Information I includes all the meta-analysis summaries for 24 cardiovascular phenotypes (blood hemoglobin level, diastolic blood pressure, heart left ventricle weight-to-body weight ratio, heart left ventricle wet weight, heart rate, heart right ventricle weight-to-left ventricle weight ratio, heart right ventricle wet weight, heart weight as percentage of body weight, heart weight-to-body weight ratio, heart wet weight, hematocrit, mean arterial blood pressure, mean corpuscular volume, plasma total cholesterol level, plasma triglyceride level, red blood cell count, serum aspartate aminotransferase activity level, serum calcium level, serum chloride level, serum free fatty acids level, serum potassium level, serum total cholesterol level, serum triglyceride level, systolic blood pressure). Information about abnormal, normal and naïve control strains for 24 phenotypes is available in Supplementary Information II.

We also created separate summaries for different age groups (age1: <70 days, age2: 70–99 days, age3: 100+ days) and gender groups if significant age or gender difference was observed in the `all age’ analysis. For example, in Figure 7, systolic blood pressure between male and female differs from each other and shows an obvious pattern (usually female has lower blood pressure). After analysis, we decided that we should produce a separate summary for both gender and for each age group as well. The same situation applies to phenotypes such as diastolic blood pressure, heart weight as percentage of body weight, heart weight-to-body weight ratio, heart rate, heart wet weight and mean arterial blood pressure. Those phenotypes have enough data for age and gender stratification while still producing meaningful meta-analysis results. Phenotypes related to ventricle weight and blood metabolite measurement suffer from lack of data for stratification (i.e. heart right ventricle weight-to-left ventricle weight ratio, heart right ventricle wet weight, plasma total cholesterol level, plasma triglyceride level, serum aspartate aminotransferase activity level, serum calcium level, serum chloride level, serum free fatty acids level and serum potassium level only have data for one age group).

Results from the meta-analysis were reviewed by a domain expert who previously had classified strains potentially within the predicted reference ranges of `normal’ and outside of `normal’ based on widespread use and characterization as control and non-control strains. Disagreements between the meta-analysis results and the domain expert’s classification were marked out. Disagreements were decided by comparing expert classification (`normal’ and outside of `normal’) and the classification results provided by our tool. The percentage of cases in which there was consistent agreement were calculated at 98% (Supplementary Information III). Some possible causes of disagreement might be (i) lack of data for certain strains, (ii) publication bias (e.g. measurements for traditionally hypertensive strains might be reported with bias toward higher blood pressure) and (iii) inherent for certain strains (strain’s phenotypes drift through multiple generations of inbreeding).

We also found that for some strains considered to exhibit `normal’ phenotypes, although the meta-analysis mean value was within the overall expected range, the upper bound or the lower bound of its individual range was beyond the overall expected range for `normal’, perhaps indicating (i) more data are needed from more institutes for more confident conclusions about individual expected ranges or (ii) variability for those strain groups may be due to potential genetic drift so that the genotypes of substrains have become more diverse or (iii) certain strains could be more susceptible to outside influences such as housing, handlers and other environmental factors on phenotypes. Good examples would be ACI_Female, BUF_Male and WAG_Female/Male/Both in Figure 7. We also found that the usual pattern between male and female (i.e. that female has lower blood pressure) sometimes did not hold true. For example, in Figure 9, the sex-specific pattern for SHRSP is opposite of the common pattern as female SHRSP rates have higher blood pressure. On the other hand, SHR exhibits the common pattern.

Summary data that stretched beyond the overall expected ranges or exhibited odd patterns generally came from meta-analyses with a limited number of experiments (usually <5). This is evidence of low confidence for the meta-analysis result, which indicates more experimental data are needed to establish a trustworthy range. This is also evidence that for meta-analysis, the number of studies included is vital to eliminate random experimental error and generate trustworthy results. The number of studies in general was more important than the number of animals in each study. Our analysis method demonstrated its potential to be used (i) to provide expected ranges for rat phenotypes and (ii) to facilitate research planning by visualizing current gaps and suggesting potential research directions to fill in the gaps. In addition, our tool can be potentially used for (i) data curation quality control (through sensitivity analysis from a forest plot result summary in Figure 7, curators could identify obviously erroneous result) and (ii) publication bias examination, which can promote better research conduct and ensure protocol consistency.

One use case resources created from this pipeline will be in the study of hypertension. Because hypertension has several subtypes, researchers might be only interested in hypertension with elevated systolic blood pressure but normal diastolic blood pressure. Researchers could refer to two expected range tables in RGD website and find that among all hypertensive strains (GH, LH, MHS, SHR and SHRSP) MHS has diastolic blood pressure close to normal range, so MHS would be a better choice in this scenario.

## Conclusion

We successfully implemented an analysis pipeline with user interface to generate expected ranges for phenotypes. The pipeline and interface provides the means to (i) identify expected ranges with customized user request, (ii) identify phenotypes without sufficient data to determine an expected range to prioritize these for acquisition through direct contact with researchers or extraction from published literature and (iii) alert RGD staff of new phenotype data in PhenoMiner and potential changes in expected range so the pipeline can be run to update the expected ranges with latest available data.

Based on the success of this project, the RGD will further develop a Precision Models Portal to present these data and link to others to provide a rich resource for investigators. The results of this study will be used to target phenotype areas for data acquisition and analysis. The goal will be to provide comprehensive profiles based on the expected ranges for phenotypes across all major physiological systems. The availability of sequence and variant data for a number of the strains will offer the opportunity to provide a complementary genotype profile with the phenotype profile to enhance the ability of researchers to choose models based on both genotype and phenotype.

## Supplementary Material

Supplemental_Information_baz037

## Notes

**Database URL**: https://rgd.mcw.edu/
