# Supplementary material for: Quantitative phenotype analysis to identify, validate and compare rat disease models
Source: Database (Oxford). 2019 Apr 2;2019:baz037. doi: 10.1093/database/baz037 (PMC6444380; doi:10.1093/database/baz037)
Supplement: Supplemental_Information_baz037 [file supplemental_information_baz037.pdf]

SUPPLEMENTARY INFORMATION I: META-ANALYSIS SUMMARIES FOR 24 CARDIOVASCULAR PHENOTYPES

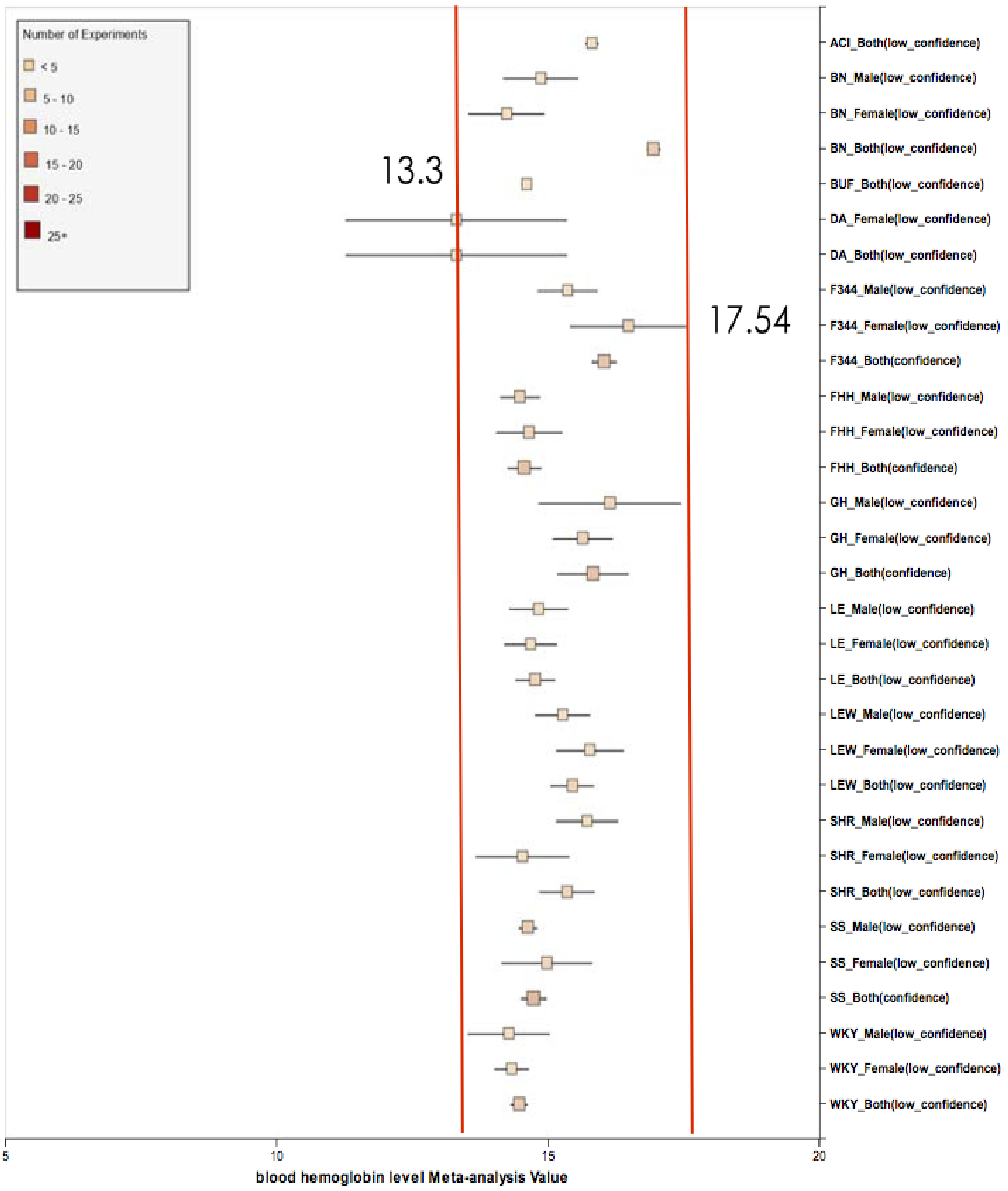

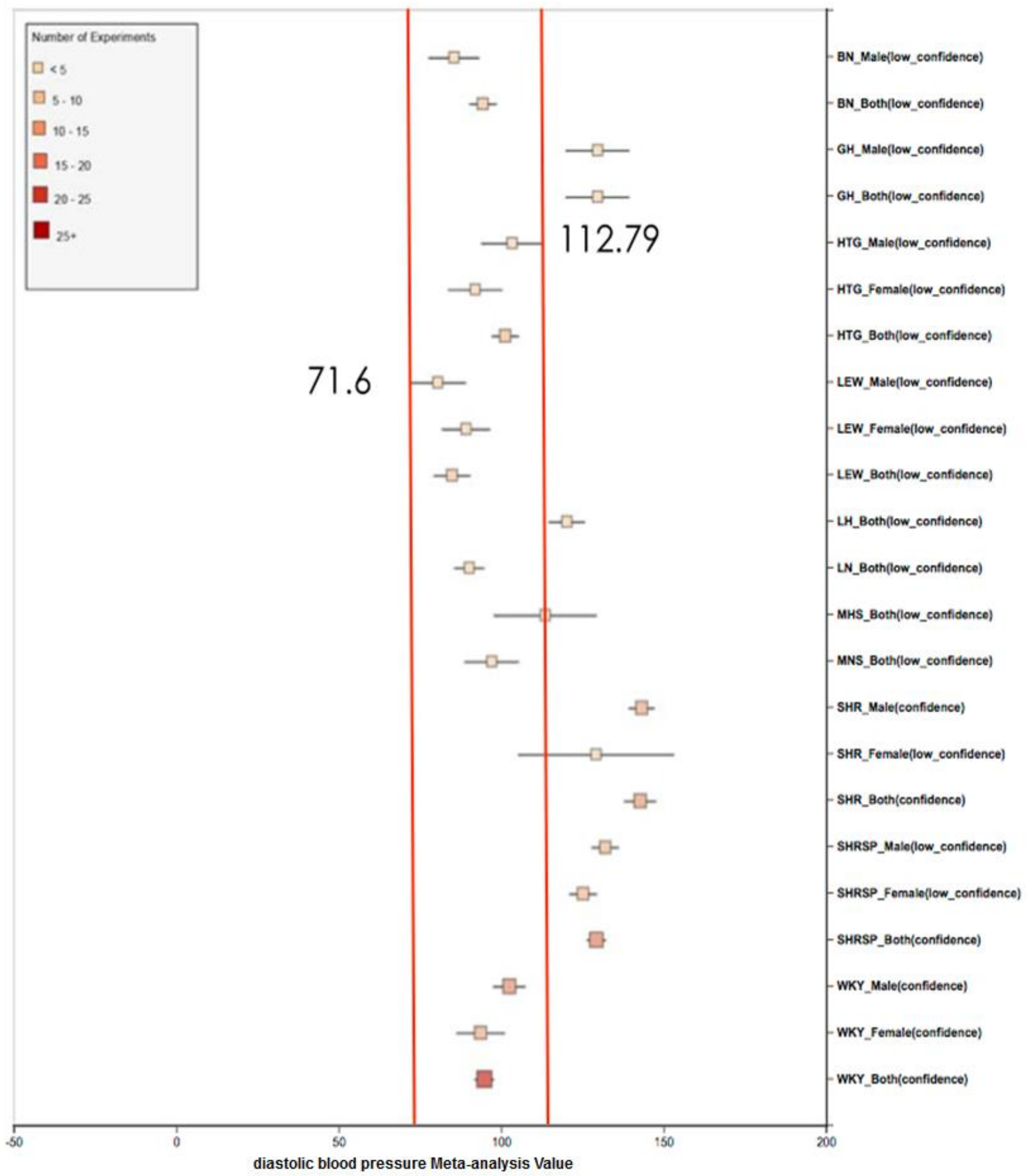

Number of Experiments
< 5
5 - 10
10 - 15
15 - 20
20 - 25
25+
112.79
71.6
BN_Male(low_confidence)
BN_Both(low_confidence)
GH_Male(low_confidence)
GH_Both(low_confidence)
HTG_Male(low_confidence)
HTG_Female(low_confidence)
HTG_Both(low_confidence)
LEW_Male(low_confidence)
LEW_Female(low_confidence)
LEW_Both(low_confidence)
LH_Both(low_confidence)
LN_Both(low_confidence)
MHS_Both(low_confidence)
MNS_Both(low_confidence)
SHR_Male(confidence)
SHR_Female(low_confidence)
SHR_Both(confidence)
SHRSP_Male(low_confidence)
SHRSP_Female(low_confidence)
SHRSP_Both(confidence)
WKY_Male(confidence)
WKY_Female(confidence)
WKY_Both(confidence)
-50
0
50
100
150
200
diastolic blood pressure Meta-analysis Value

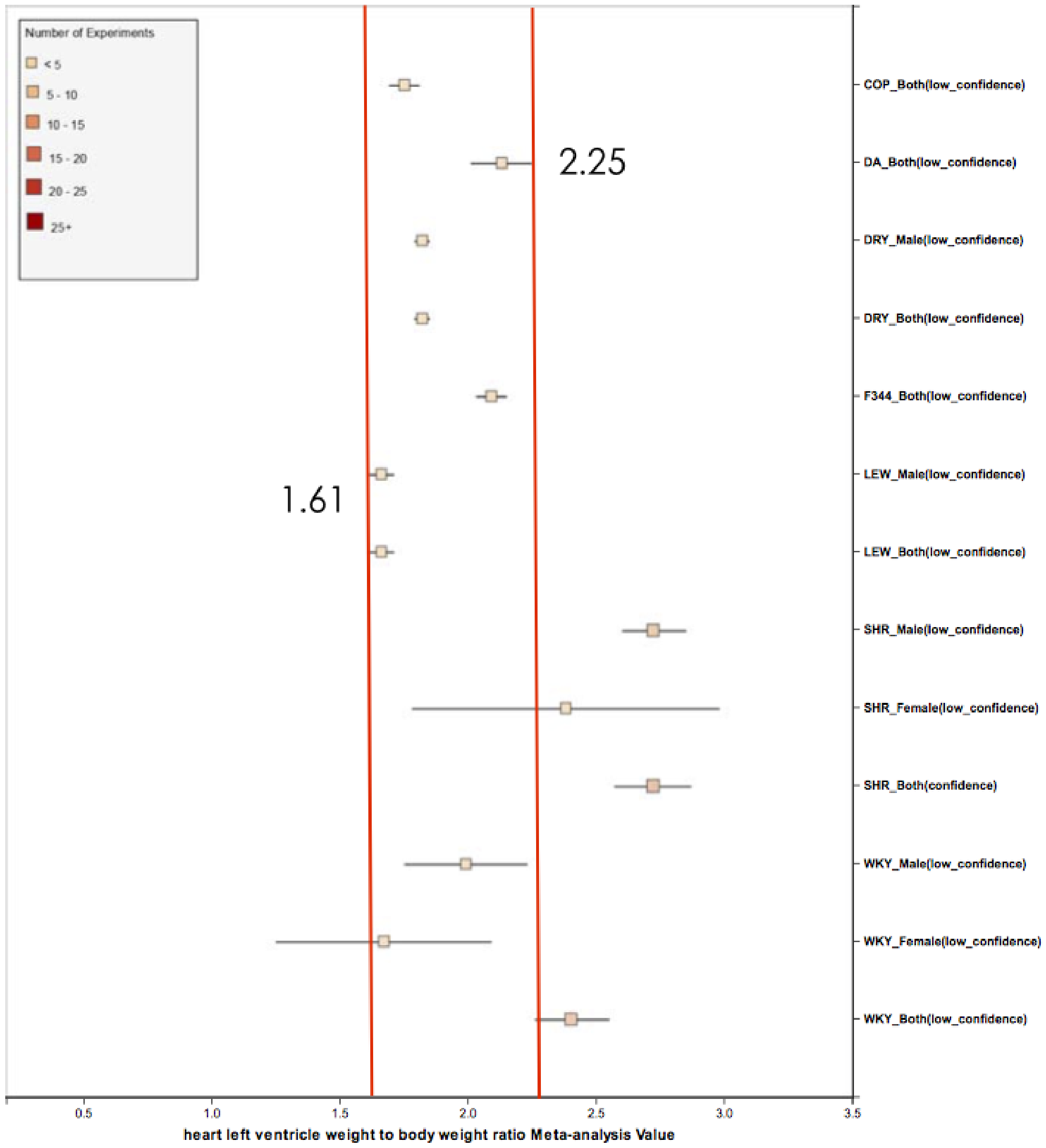
Number of Experiments
< 5
5 - 10
10 - 15
15 - 20
20 - 25
25+
2.25
1.61
COP_Both(low_confidence)
DA_Both(low_confidence)
DRY_Male(low_confidence)
DRY_Both(low_confidence)
F344_Both(low_confidence)
LEW_Male(low_confidence)
LEW_Both(low_confidence)
SHR_Male(low_confidence)
SHR_Female(low_confidence)
SHR_Both(confidence)
WKY_Male(low_confidence)
WKY_Female(low_confidence)
WKY_Both(low_confidence)
0.5
1.0
1.5
2.0
2.5
3.0
3.5
heart left ventricle weight to body weight ratio Meta-analysis Value

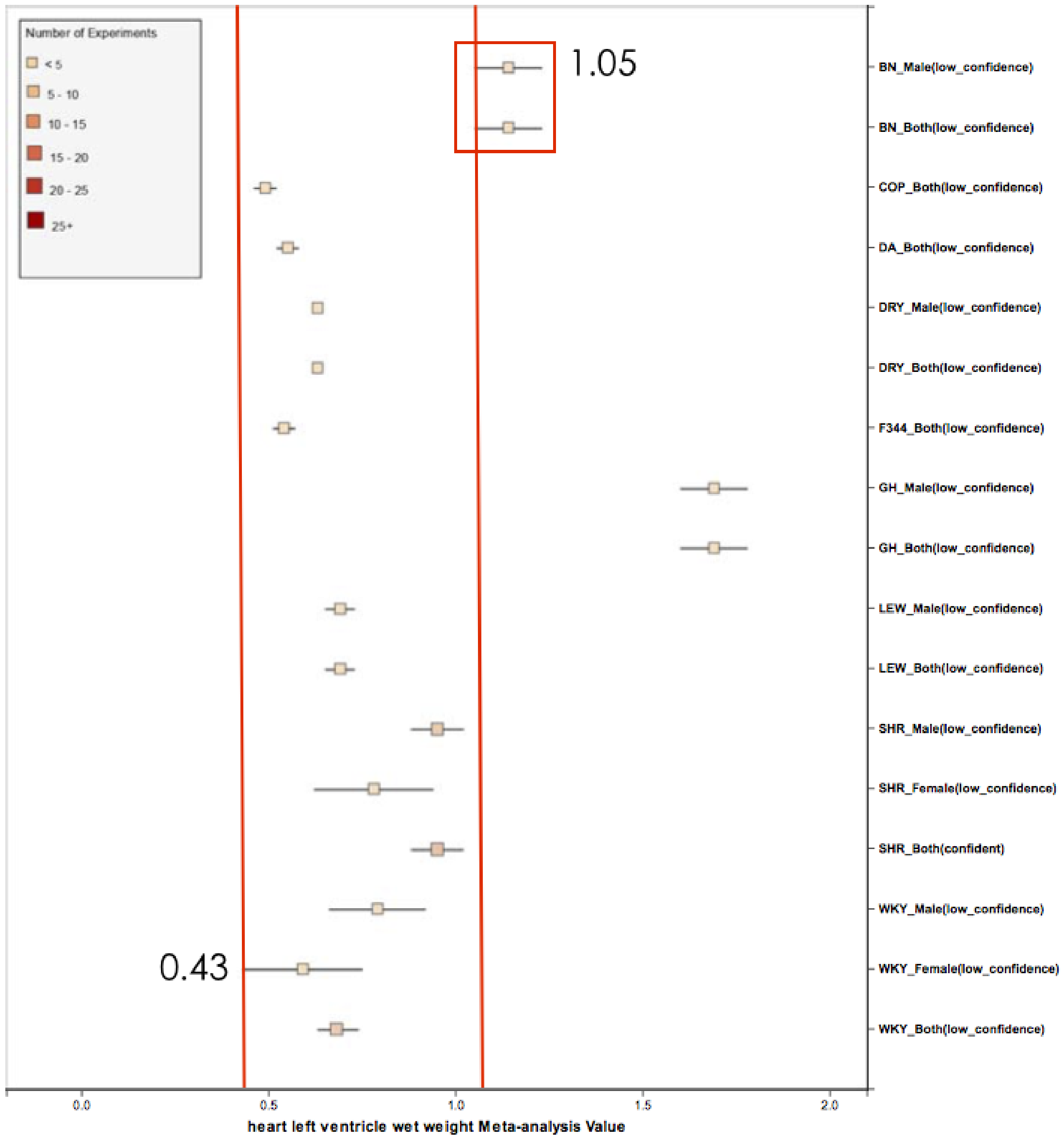

Number of Experiments
< 5
5 - 10
10 - 15
15 - 20
20 - 25
25+
1.05
0.43
BN_Male(low_confidence)
BN_Both(low_confidence)
COP_Both(low_confidence)
DA_Both(low_confidence)
DRY_Male(low_confidence)
DRY_Both(low_confidence)
F344_Both(low_confidence)
GH_Male(low_confidence)
GH_Both(low_confidence)
LEW_Male(low_confidence)
LEW_Both(low_confidence)
SHR_Male(low_confidence)
SHR_Female(low_confidence)
SHR_Both(confident)
WKY_Male(low_confidence)
WKY_Female(low_confidence)
WKY_Both(low_confidence)
0.0
0.5
1.0
1.5
2.0
heart left ventricle wet weight Meta-analysis Value

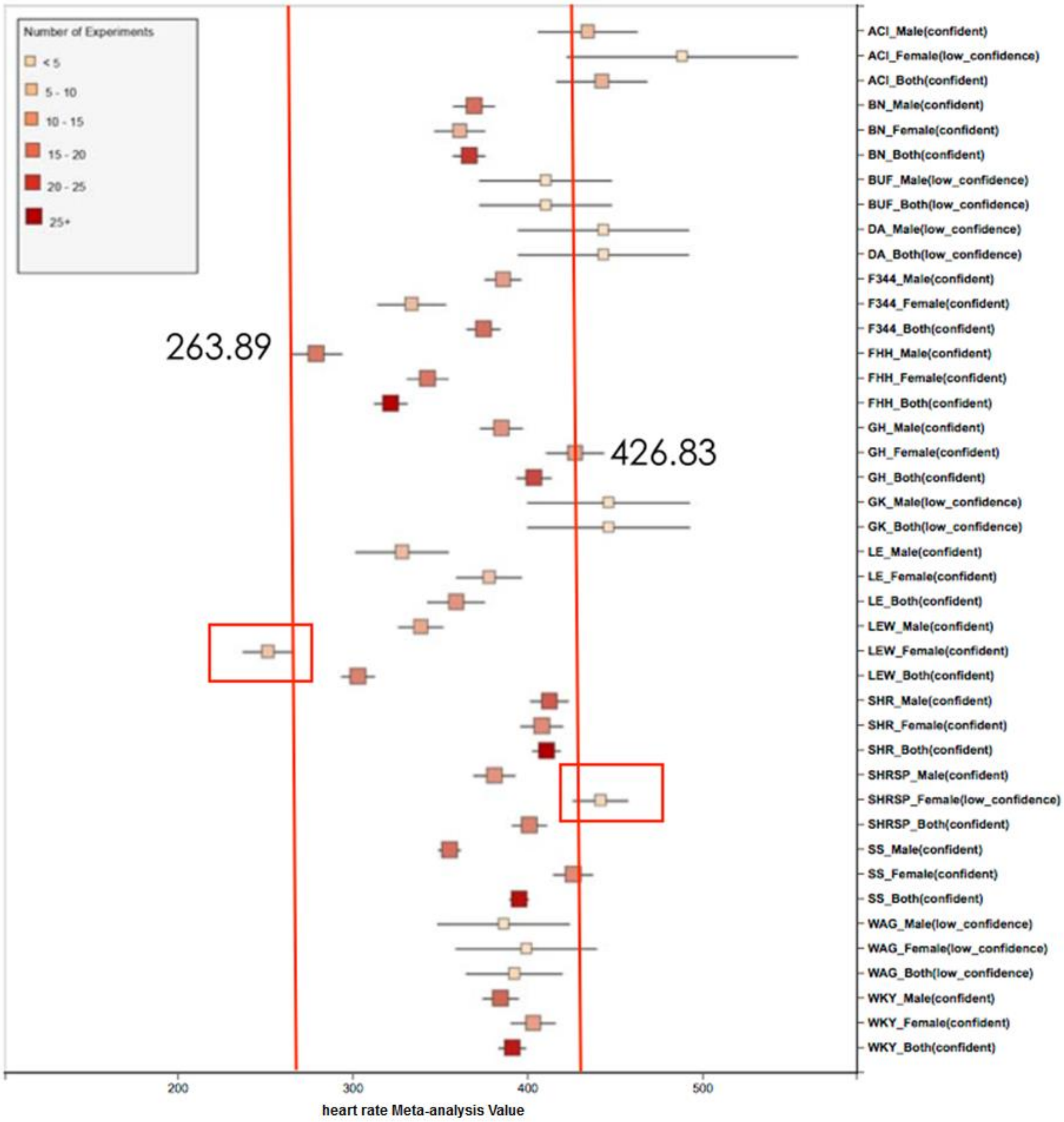

Number of Experiments
< 5
5 - 10
10 - 15
15 - 20
20 - 25
25+
263.89
426.83
ACI_Male(confident)
ACI_Female(low_confidence)
ACI_Both(confident)
BN_Male(confident)
BN_Female(confident)
BN_Both(confident)
BUF_Male(low_confidence)
BUF_Both(low_confidence)
DA_Male(low_confidence)
DA_Both(low_confidence)
F344_Male(confident)
F344_Female(confident)
F344_Both(confident)
FHH_Male(confident)
FHH_Female(confident)
FHH_Both(confident)
GH_Male(confident)
GH_Female(confident)
GH_Both(confident)
GK_Male(low_confidence)
GK_Both(low_confidence)
LE_Male(confident)
LE_Female(confident)
LE_Both(confident)
LEW_Male(confident)
LEW_Female(confident)
LEW_Both(confident)
SHR_Male(confident)
SHR_Female(confident)
SHR_Both(confident)
SHRSP_Male(confident)
SHRSP_Female(low_confidence)
SHRSP_Both(confident)
SS_Male(confident)
SS_Female(confident)
SS_Both(confident)
WAG_Male(low_confidence)
WAG_Female(low_confidence)
WAG_Both(low_confidence)
WKY_Male(confident)
WKY_Female(confident)
WKY_Both(confident)
200
300
400
500
heart rate Meta-analysis Value

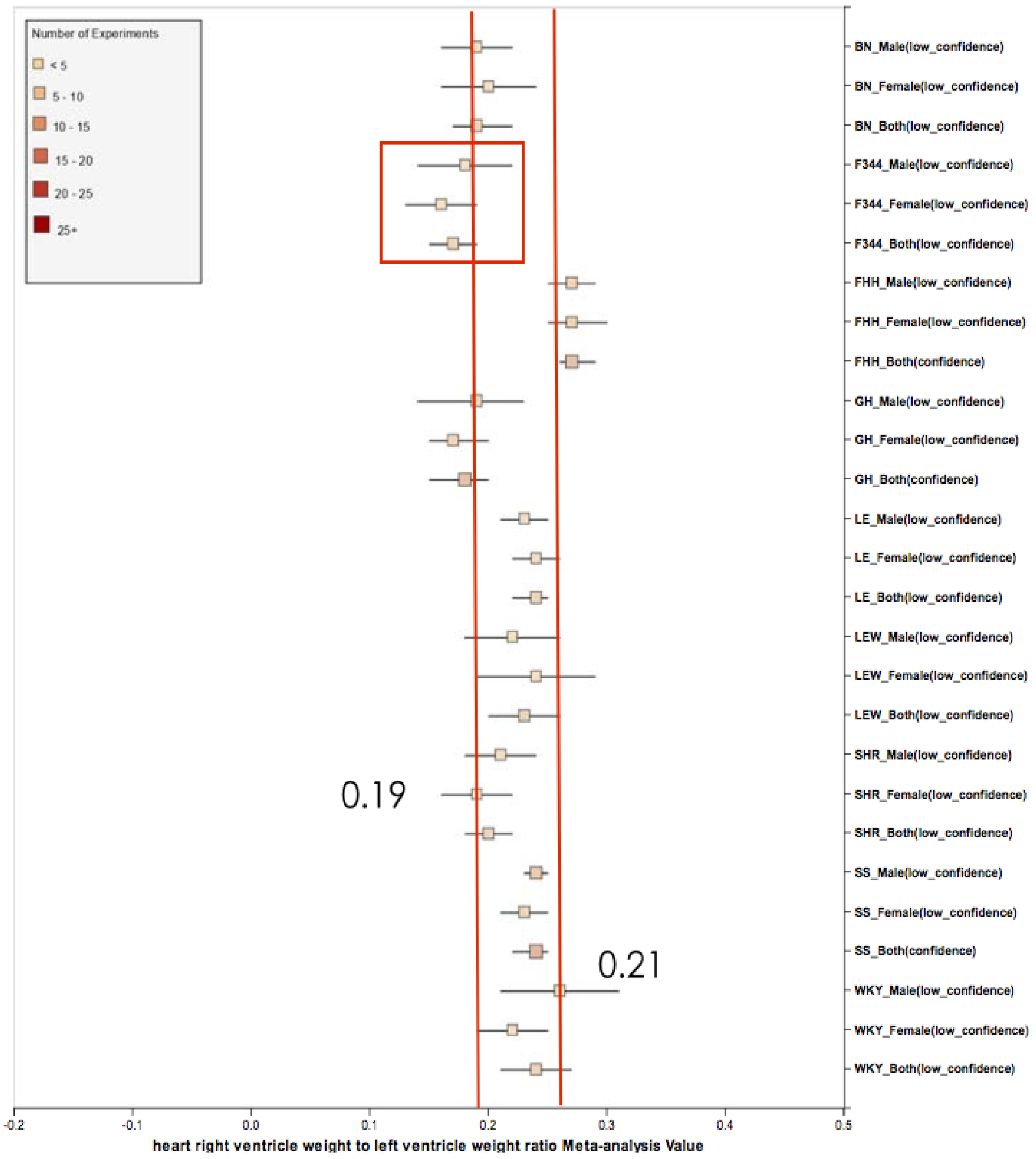

Number of Experiments
< 5
5 - 10
10 - 15
15 - 20
20 - 25
25+
BN_Male(low_confidence)
BN_Female(low_confidence)
BN_Both(low_confidence)
F344_Male(low_confidence)
F344_Female(low_confidence)
F344_Both(low_confidence)
FHH_Male(low_confidence)
FHH_Female(low_confidence)
FHH_Both(confidence)
GH_Male(low_confidence)
GH_Female(low_confidence)
GH_Both(confidence)
LE_Male(low_confidence)
LE_Female(low_confidence)
LE_Both(low_confidence)
LEW_Male(low_confidence)
LEW_Female(low_confidence)
LEW_Both(low_confidence)
SHR_Male(low_confidence)
SHR_Female(low_confidence)
SHR_Both(low_confidence)
SS_Male(low_confidence)
SS_Female(low_confidence)
SS_Both(confidence)
WKY_Male(low_confidence)
WKY_Female(low_confidence)
WKY_Both(low_confidence)
0.19
0.21
-0.2
-0.1
0.0
0.1
0.2
0.3
0.4
0.5
heart right ventricle weight to left ventricle weight ratio Meta-analysis Value

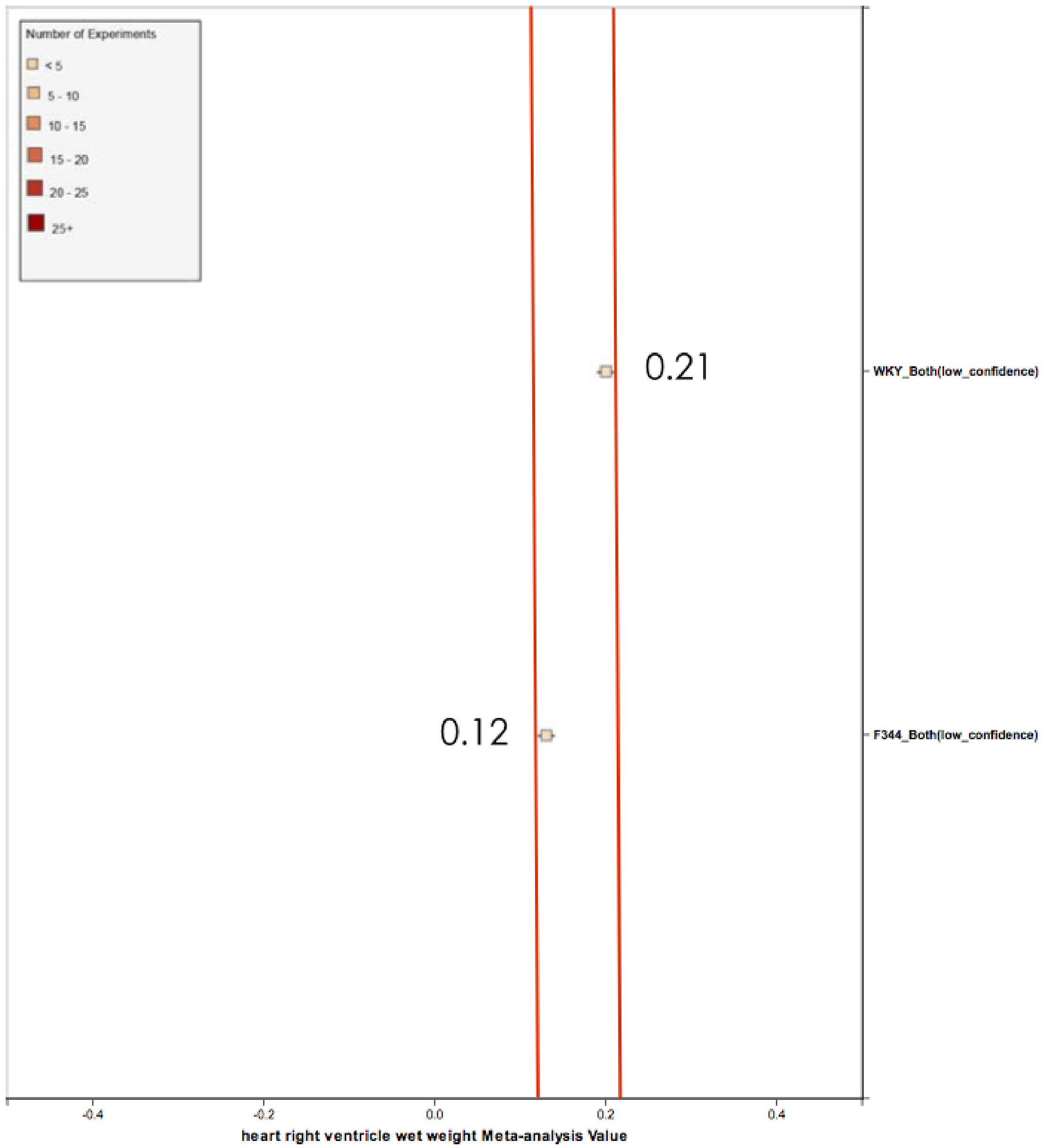
Number of Experiments
< 5
5 - 10
10 - 15
15 - 20
20 - 25
25+
0.21
0.12
WKY_Both(low_confidence)
F344_Both(low_confidence)
-0.4
-0.2
0.0
0.2
0.4
heart right ventricle wet weight Meta-analysis Value

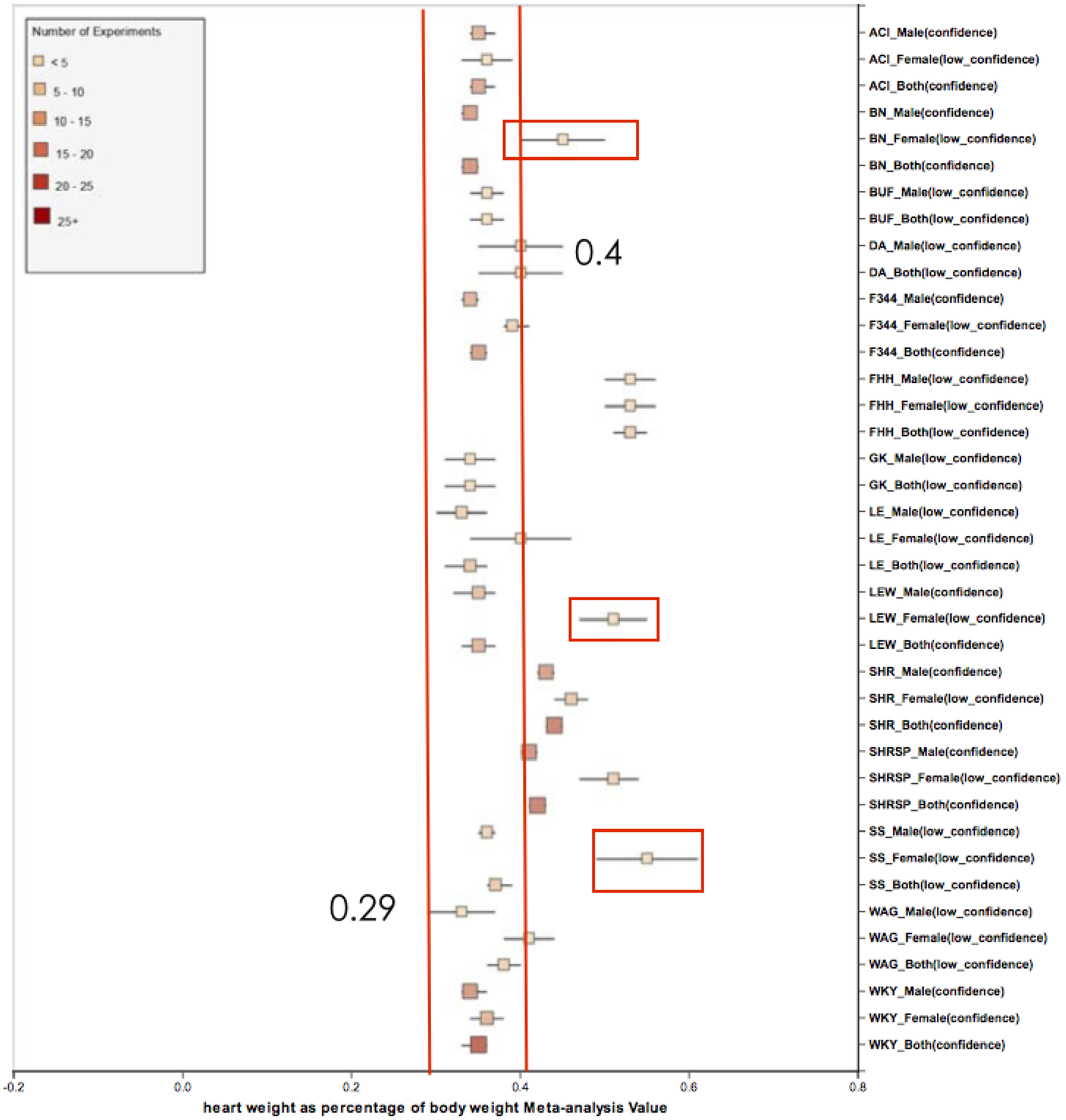

Number of Experiments
< 5
5 - 10
10 - 15
15 - 20
20 - 25
25+
0.4
0.29
ACI_Male(confidence)
ACI_Female(low_confidence)
ACI_Both(confidence)
BN_Male(confidence)
BN_Female(low_confidence)
BN_Both(confidence)
BUF_Male(low_confidence)
BUF_Both(low_confidence)
DA_Male(low_confidence)
DA_Both(low_confidence)
F344_Male(confidence)
F344_Female(low_confidence)
F344_Both(confidence)
FHH_Male(low_confidence)
FHH_Female(low_confidence)
FHH_Both(low_confidence)
GK_Male(low_confidence)
GK_Both(low_confidence)
LE_Male(low_confidence)
LE_Female(low_confidence)
LE_Both(low_confidence)
LEW_Male(confidence)
LEW_Female(low_confidence)
LEW_Both(confidence)
SHR_Male(confidence)
SHR_Female(low_confidence)
SHR_Both(confidence)
SHRSP_Male(confidence)
SHRSP_Female(low_confidence)
SHRSP_Both(confidence)
SS_Male(low_confidence)
SS_Female(low_confidence)
SS_Both(low_confidence)
WAG_Male(low_confidence)
WAG_Female(low_confidence)
WAG_Both(low_confidence)
WKY_Male(confidence)
WKY_Female(confidence)
WKY_Both(confidence)
-0.2
0.0
0.2
0.4
0.6
0.8
heart weight as percentage of body weight Meta-analysis Value

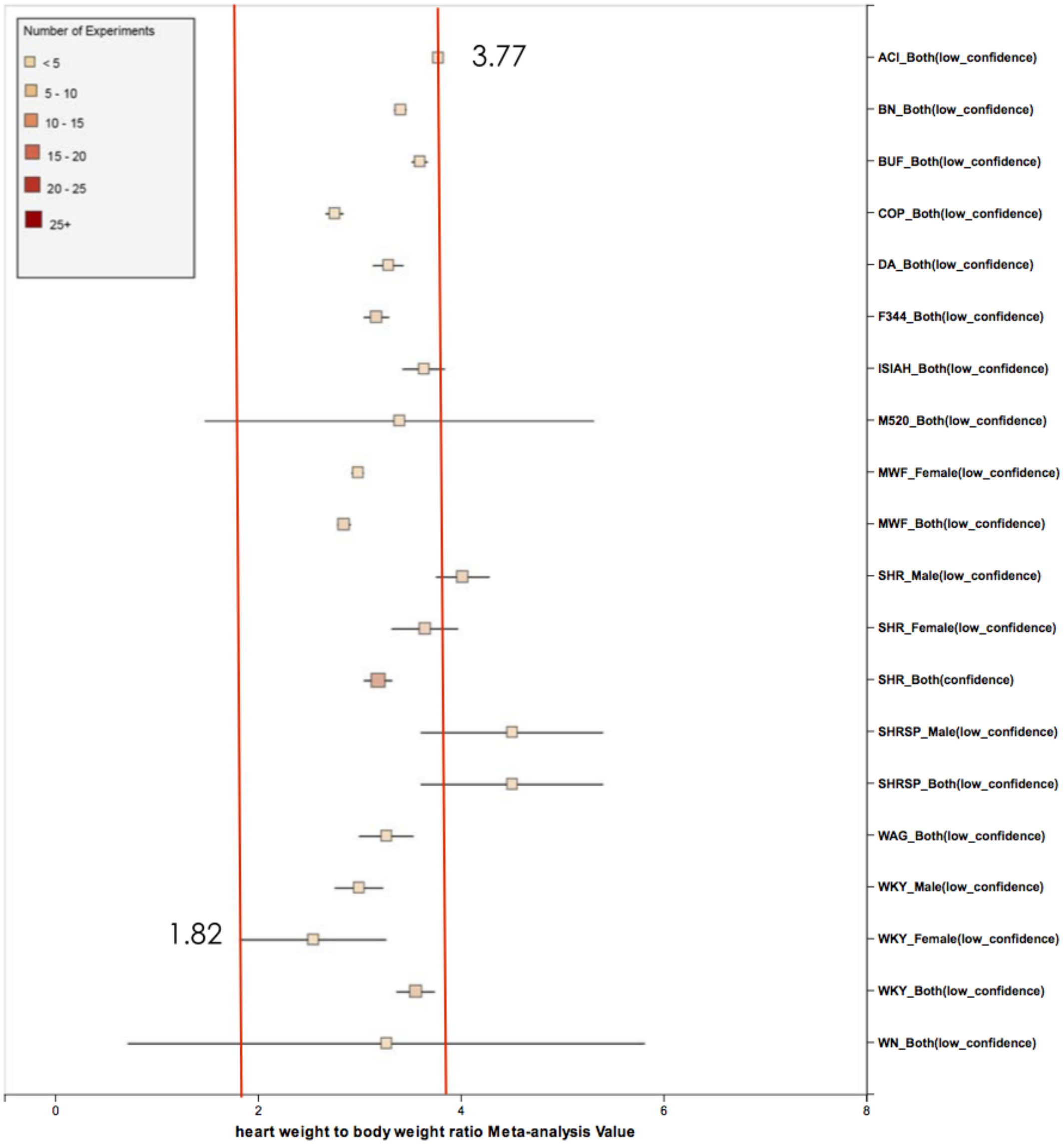

Number of Experiments
< 5
5 - 10
10 - 15
15 - 20
20 - 25
25+
3.77
1.82
ACI_Both(low_confidence)
BN_Both(low_confidence)
BUF_Both(low_confidence)
COP_Both(low_confidence)
DA_Both(low_confidence)
F344_Both(low_confidence)
ISIAH_Both(low_confidence)
M520_Both(low_confidence)
MWF_Female(low_confidence)
MWF_Both(low_confidence)
SHR_Male(low_confidence)
SHR_Female(low_confidence)
SHR_Both(confidence)
SHRSP_Male(low_confidence)
SHRSP_Both(low_confidence)
WAG_Both(low_confidence)
WKY_Male(low_confidence)
WKY_Female(low_confidence)
WKY_Both(low_confidence)
WN_Both(low_confidence)
0
2
4
6
8
heart weight to body weight ratio Meta-analysis Value

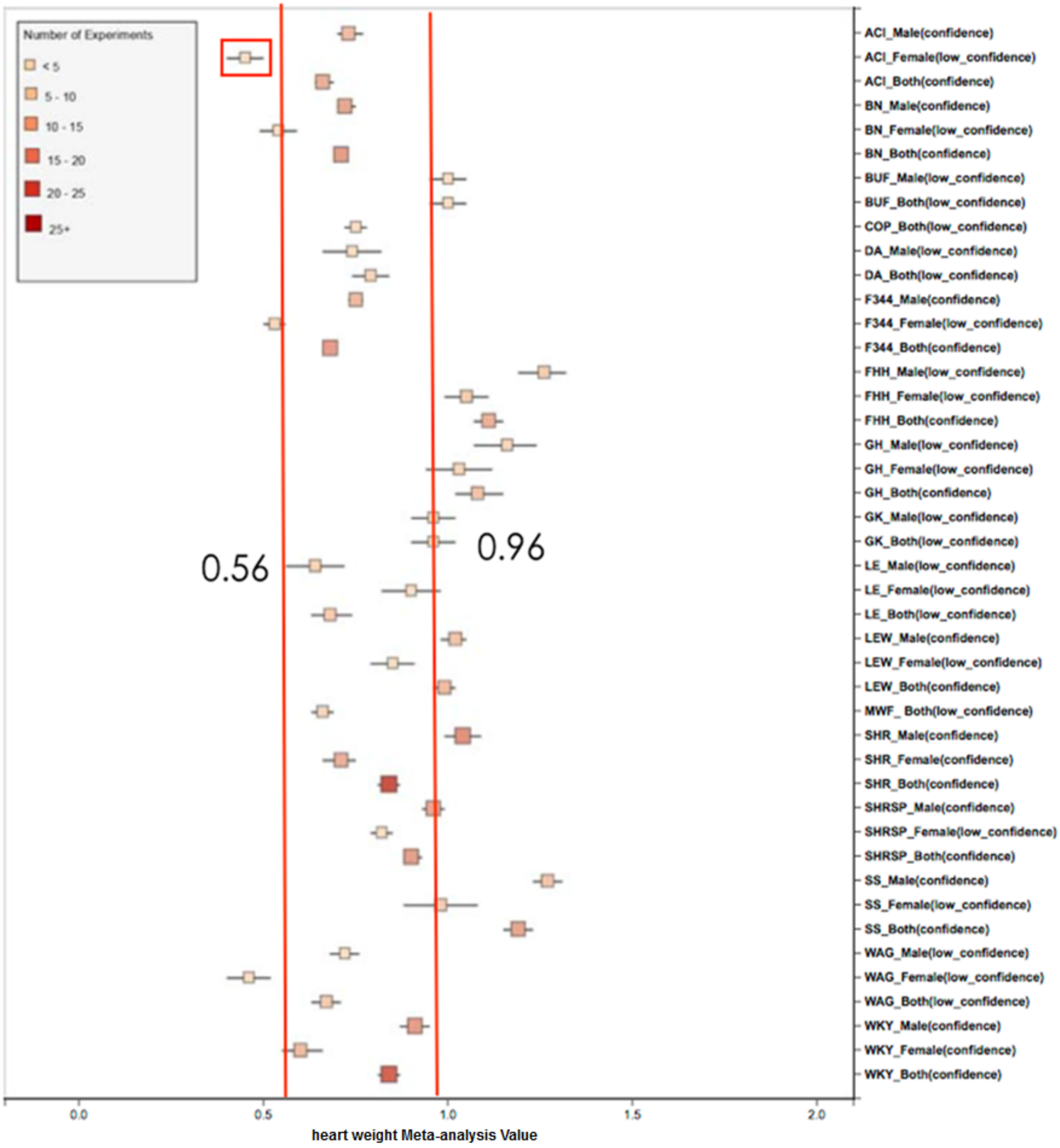
Number of Experiments
< 5
5 - 10
10 - 15
15 - 20
20 - 25
25+
0.56
0.96
ACI_Male(confidence)
ACI_Female(low_confidence)
ACI_Both(confidence)
BN_Male(confidence)
BN_Female(low_confidence)
BN_Both(confidence)
BUF_Male(low_confidence)
BUF_Both(low_confidence)
COP_Both(low_confidence)
DA_Male(low_confidence)
DA_Both(low_confidence)
F344_Male(confidence)
F344_Female(low_confidence)
F344_Both(confidence)
FHH_Male(low_confidence)
FHH_Female(low_confidence)
FHH_Both(confidence)
GH_Male(low_confidence)
GH_Female(low_confidence)
GH_Both(confidence)
GK_Male(low_confidence)
GK_Both(low_confidence)
LE_Male(low_confidence)
LE_Female(low_confidence)
LE_Both(low_confidence)
LEW_Male(confidence)
LEW_Female(low_confidence)
LEW_Both(confidence)
MWF_ Both(low_confidence)
SHR_Male(confidence)
SHR_Female(confidence)
SHR_Both(confidence)
SHRSP_Male(confidence)
SHRSP_Female(low_confidence)
SHRSP_Both(confidence)
SS_Male(confidence)
SS_Female(low_confidence)
SS_Both(confidence)
WAG_Male(low_confidence)
WAG_Female(low_confidence)
WAG_Both(low_confidence)
WKY_Male(confidence)
WKY_Female(confidence)
WKY_Both(confidence)
0.0
0.5
1.0
1.5
2.0
heart weight Meta-analysis Value

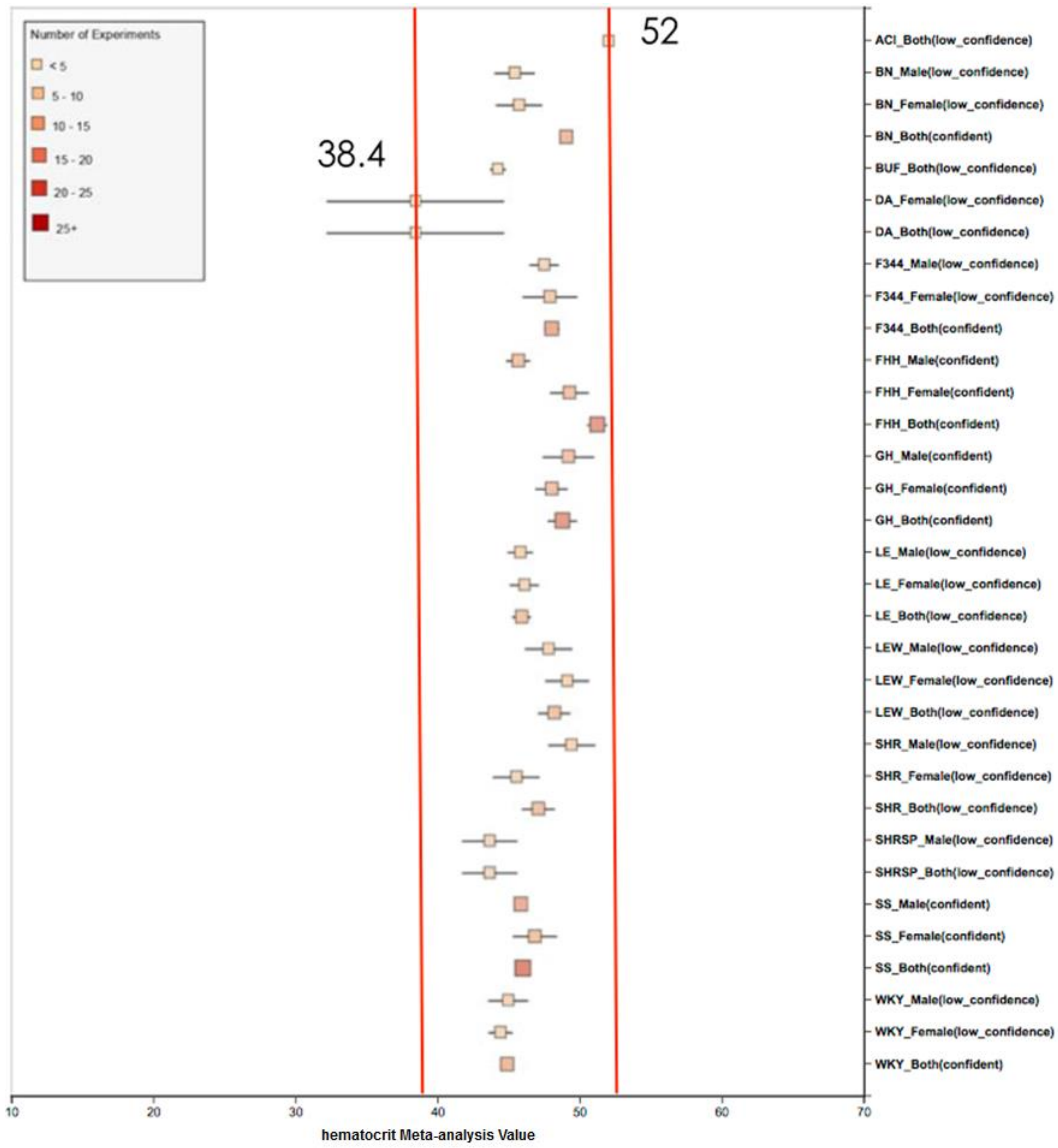

Number of Experiments
< 5
5 - 10
10 - 15
15 - 20
20 - 25
25+
52
38.4
ACI_Both(low_confidence)
BN_Male(low_confidence)
BN_Female(low_confidence)
BN_Both(confident)
BUF_Both(low_confidence)
DA_Female(low_confidence)
DA_Both(low_confidence)
F344_Male(low_confidence)
F344_Female(low_confidence)
F344_Both(confident)
FHH_Male(confident)
FHH_Female(confident)
FHH_Both(confident)
GH_Male(confident)
GH_Female(confident)
GH_Both(confident)
LE_Male(low_confidence)
LE_Female(low_confidence)
LE_Both(low_confidence)
LEW_Male(low_confidence)
LEW_Female(low_confidence)
LEW_Both(low_confidence)
SHR_Male(low_confidence)
SHR_Female(low_confidence)
SHR_Both(low_confidence)
SHRSP_Male(low_confidence)
SHRSP_Both(low_confidence)
SS_Male(confident)
SS_Female(confident)
SS_Both(confident)
WKY_Male(low_confidence)
WKY_Female(low_confidence)
WKY_Both(confident)
10
20
30
40
50
60
70
hematocrit Meta-analysis Value

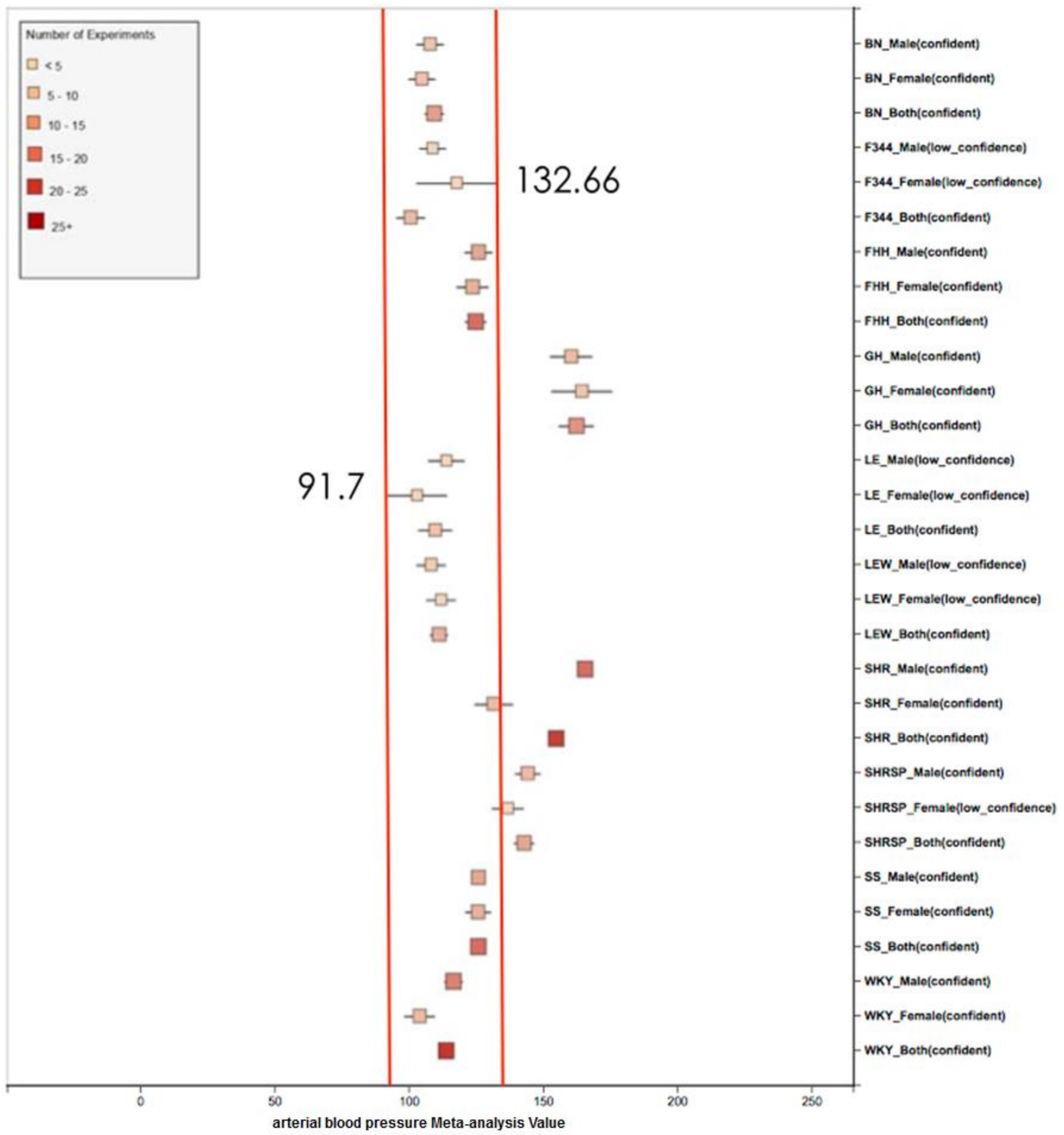

Number of Experiments
< 5
5 - 10
10 - 15
15 - 20
20 - 25
25+
132.66
91.7
BN_Male(confident)
BN_Female(confident)
BN_Both(confident)
F344_Male(low_confidence)
F344_Female(low_confidence)
F344_Both(confident)
FHH_Male(confident)
FHH_Female(confident)
FHH_Both(confident)
GH_Male(confident)
GH_Female(confident)
GH_Both(confident)
LE_Male(low_confidence)
LE_Female(low_confidence)
LE_Both(confident)
LEW_Male(low_confidence)
LEW_Female(low_confidence)
LEW_Both(confident)
SHR_Male(confident)
SHR_Female(confident)
SHR_Both(confident)
SHRSP_Male(confident)
SHRSP_Female(low_confidence)
SHRSP_Both(confident)
SS_Male(confident)
SS_Female(confident)
SS_Both(confident)
WKY_Male(confident)
WKY_Female(confident)
WKY_Both(confident)
0
50
100
150
200
250
arterial blood pressure Meta-analysis Value

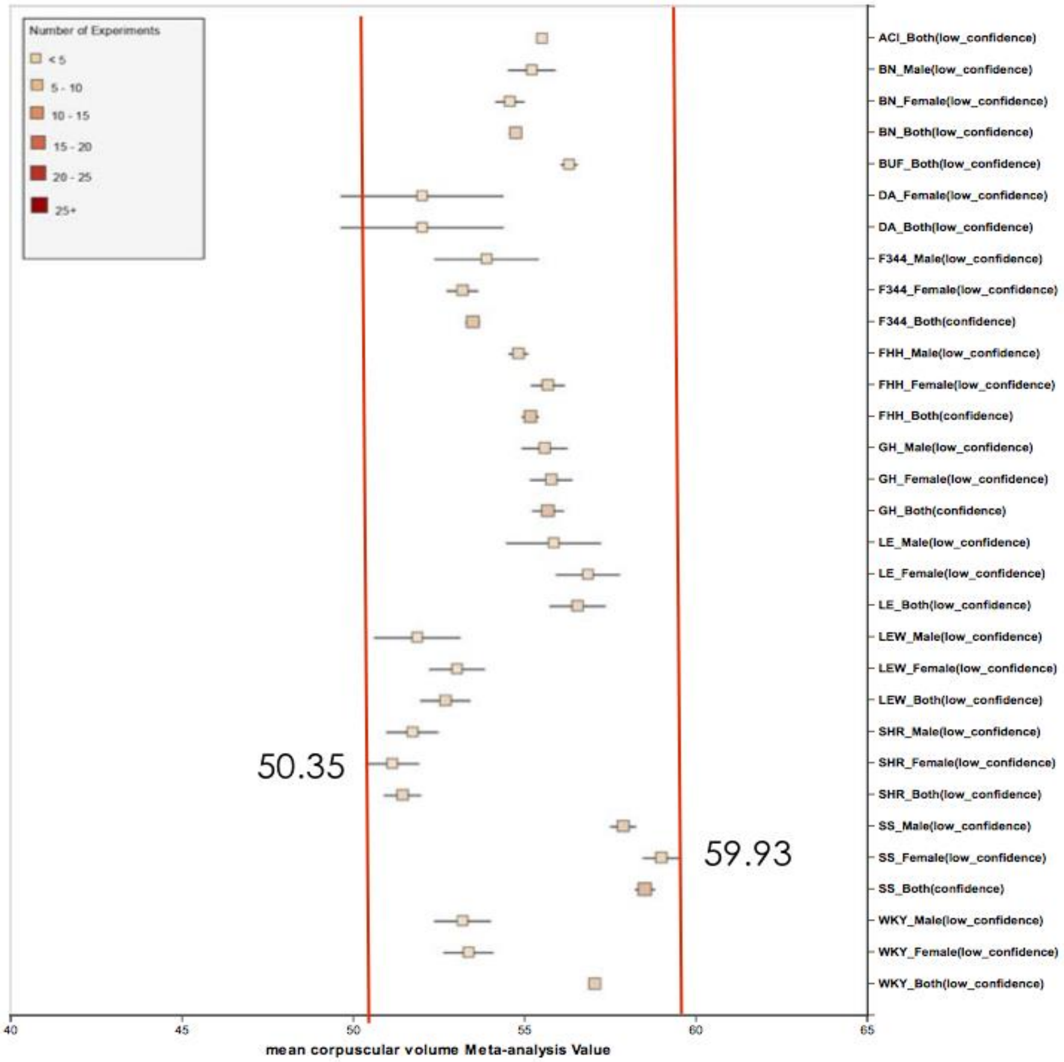

Number of Experiments
< 5
5 - 10
10 - 15
15 - 20
20 - 25
25+
ACI_Both(low_confidence)
BN_Male(low_confidence)
BN_Female(low_confidence)
BN_Both(low_confidence)
BUF_Both(low_confidence)
DA_Female(low_confidence)
DA_Both(low_confidence)
F344_Male(low_confidence)
F344_Female(low_confidence)
F344_Both(confidence)
FHH_Male(low_confidence)
FHH_Female(low_confidence)
FHH_Both(confidence)
GH_Male(low_confidence)
GH_Female(low_confidence)
GH_Both(confidence)
LE_Male(low_confidence)
LE_Female(low_confidence)
LE_Both(low_confidence)
LEW_Male(low_confidence)
LEW_Female(low_confidence)
LEW_Both(low_confidence)
SHR_Male(low_confidence)
SHR_Female(low_confidence)
SHR_Both(low_confidence)
SS_Male(low_confidence)
SS_Female(low_confidence)
SS_Both(confidence)
WKY_Male(low_confidence)
WKY_Female(low_confidence)
WKY_Both(low_confidence)
50.35
59.93
40
45
50
55
60
65
mean corpuscular volume Meta-analysis Value

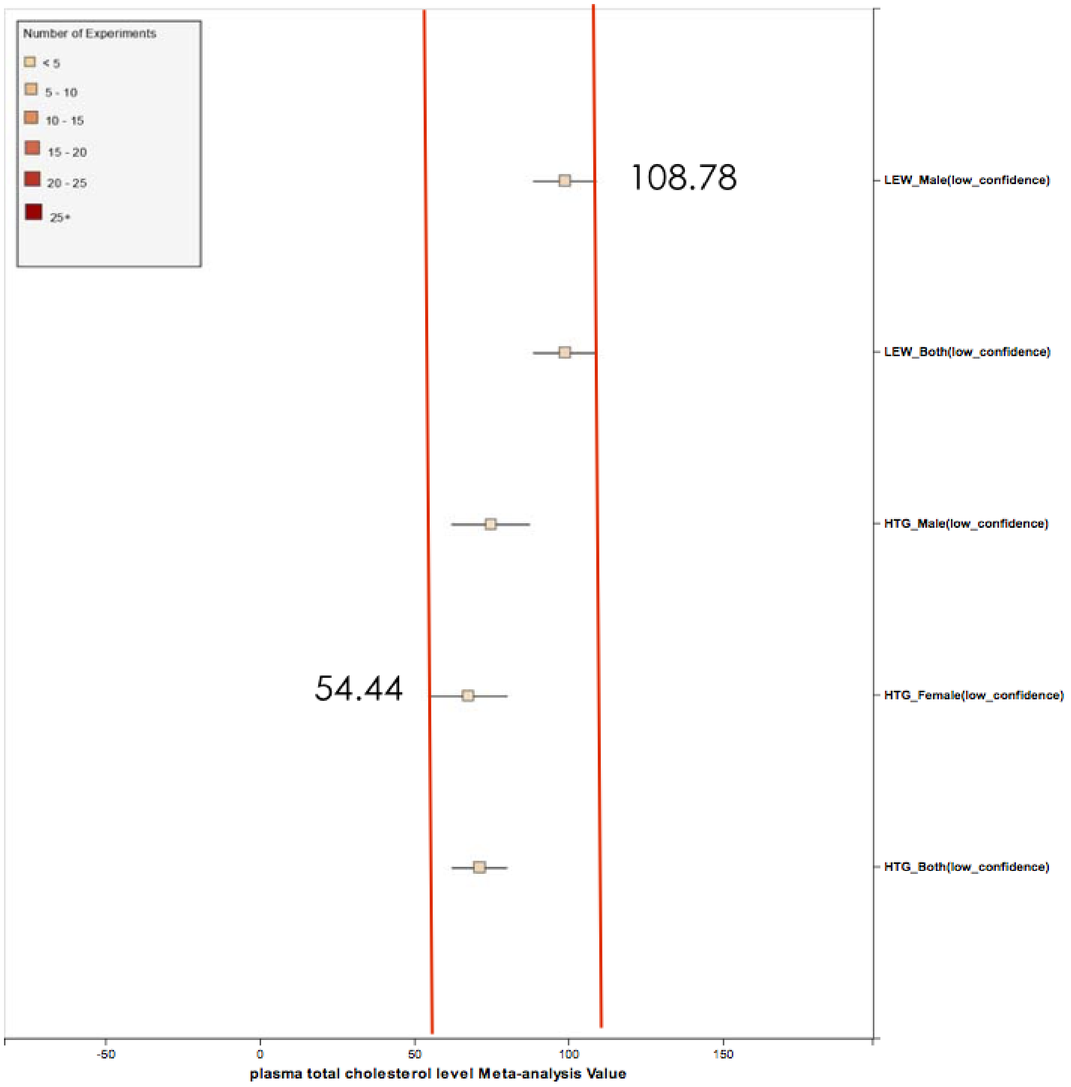

Number of Experiments
< 5
5 - 10
10 - 15
15 - 20
20 - 25
25+
108.78
54.44
LEW_Male(low_confidence)
LEW_Both(low_confidence)
HTG_Male(low_confidence)
HTG_Female(low_confidence)
HTG_Both(low_confidence)
-50
0
50
100
150
plasma total cholesterol level Meta-analysis Value

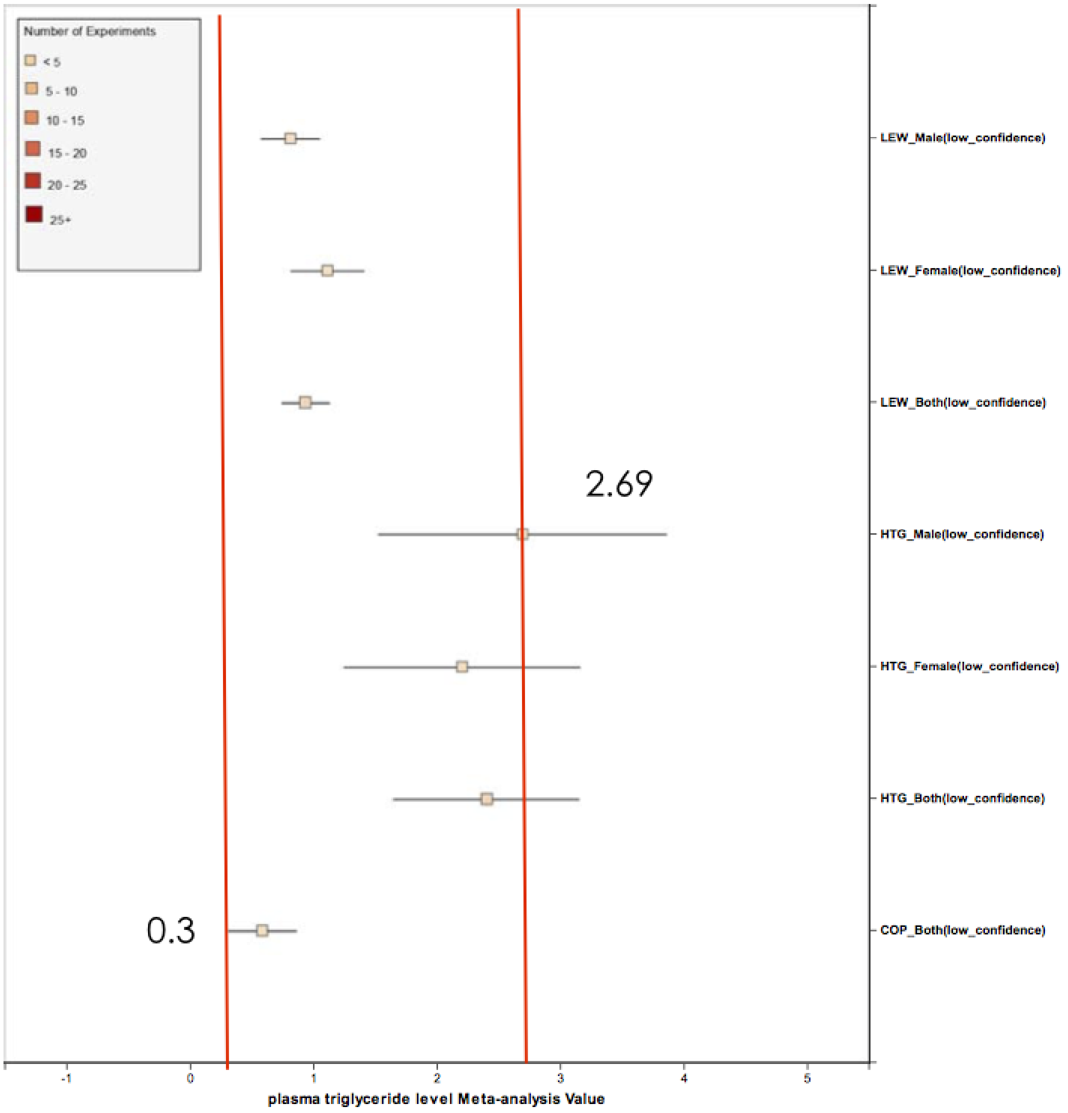
Number of Experiments
< 5
5 - 10
10 - 15
15 - 20
20 - 25
25+
LEW_Male(low_confidence)
LEW_Female(low_confidence)
LEW_Both(low_confidence)
2.69
HTG_Male(low_confidence)
HTG_Female(low_confidence)
HTG_Both(low_confidence)
0.3
COP_Both(low_confidence)
-1
0
1
2
3
4
5
plasma triglyceride level Meta-analysis Value

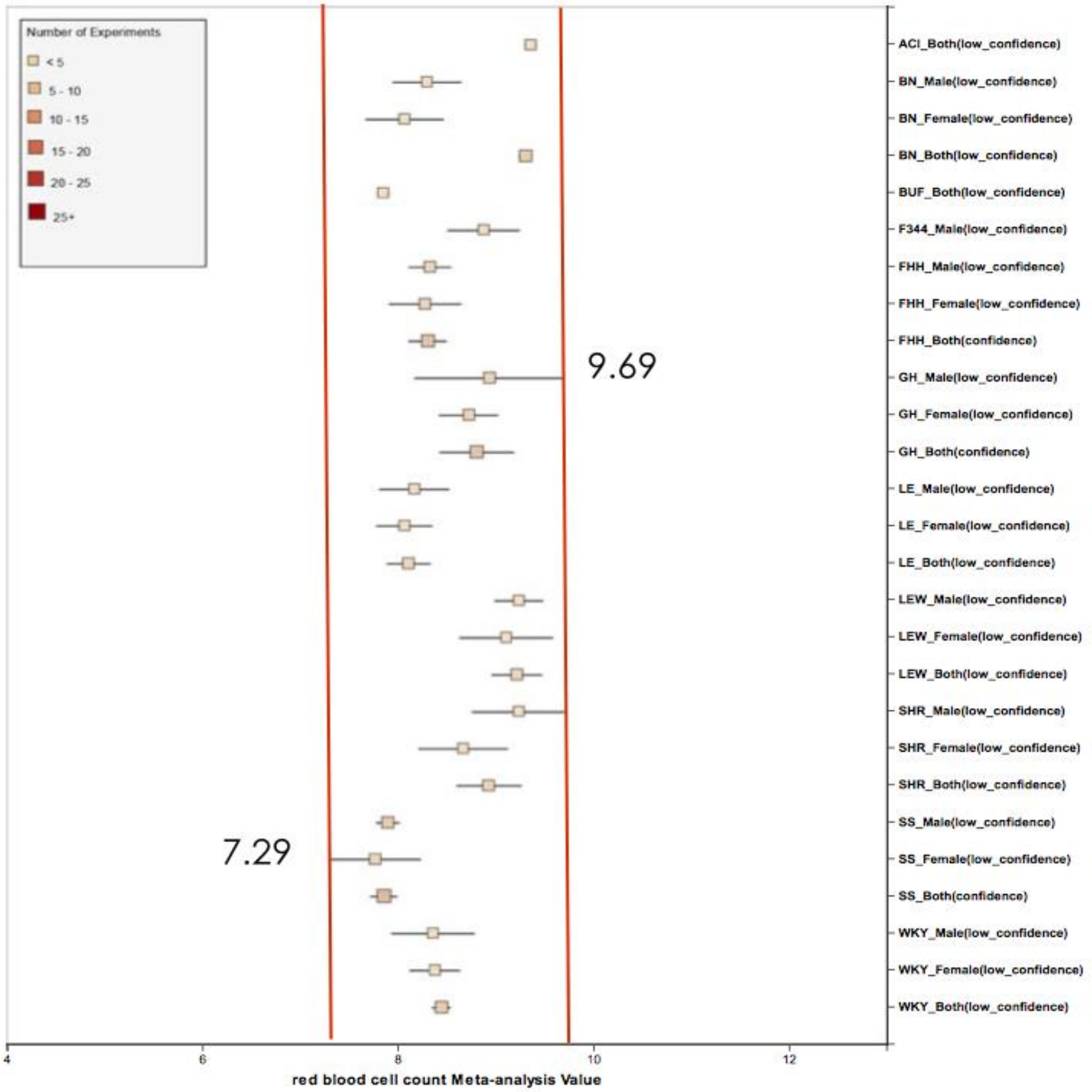

Number of Experiments
< 5
5 - 10
10 - 15
15 - 20
20 - 25
25+
9.69
7.29
ACI_Both(low_confidence)
BN_Male(low_confidence)
BN_Female(low_confidence)
BN_Both(low_confidence)
BUF_Both(low_confidence)
F344_Male(low_confidence)
FHH_Male(low_confidence)
FHH_Female(low_confidence)
FHH_Both(confidence)
GH_Male(low_confidence)
GH_Female(low_confidence)
GH_Both(confidence)
LE_Male(low_confidence)
LE_Female(low_confidence)
LE_Both(low_confidence)
LEW_Male(low_confidence)
LEW_Female(low_confidence)
LEW_Both(low_confidence)
SHR_Male(low_confidence)
SHR_Female(low_confidence)
SHR_Both(low_confidence)
SS_Male(low_confidence)
SS_Female(low_confidence)
SS_Both(confidence)
WKY_Male(low_confidence)
WKY_Female(low_confidence)
WKY_Both(low_confidence)
4
6
8
10
12
red blood cell count Meta-analysis Value

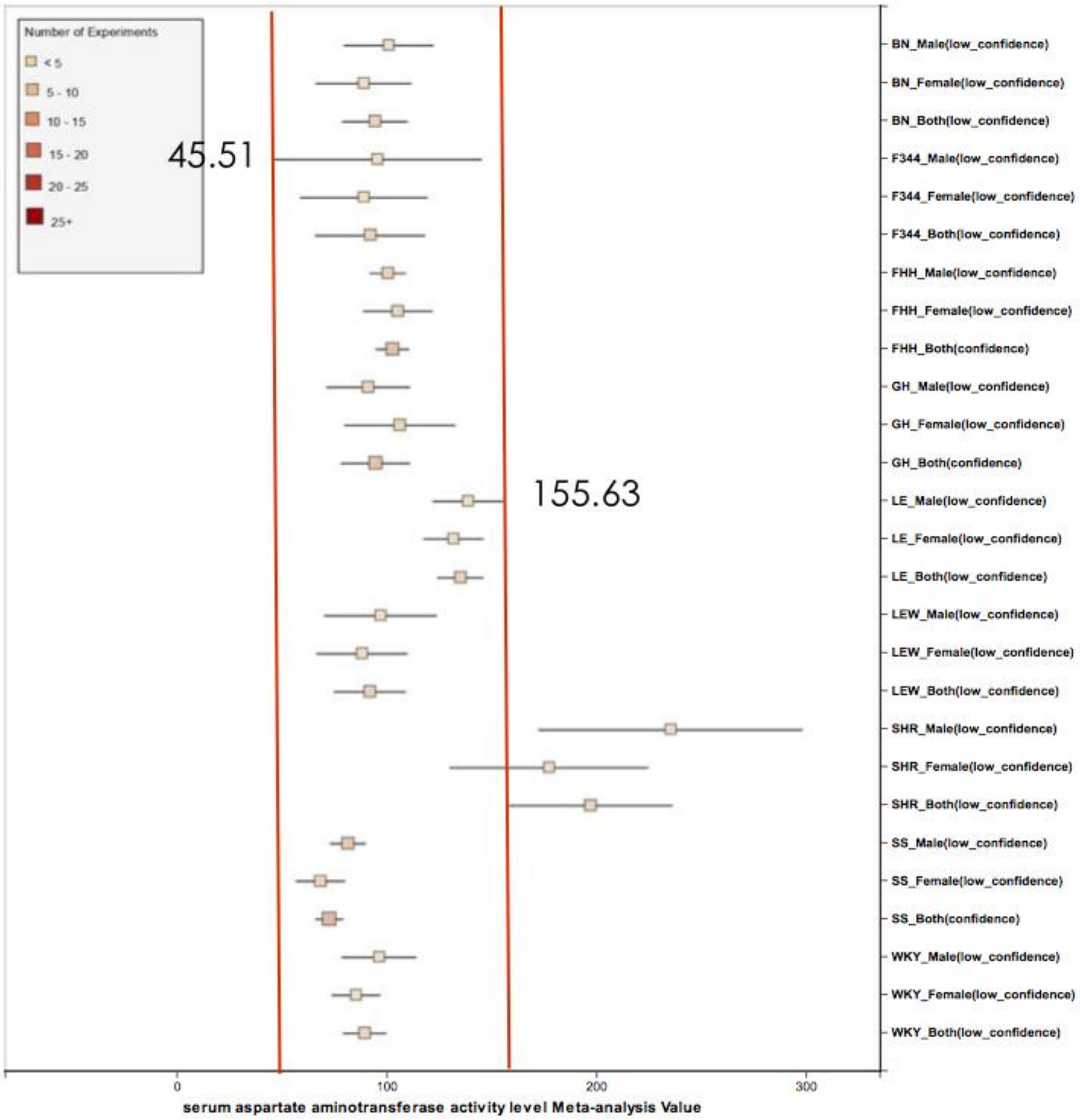

Number of Experiments
< 5
5 - 10
10 - 15
15 - 20
20 - 25
25+
45.51
155.63
BN_Male(low_confidence)
BN_Female(low_confidence)
BN_Both(low_confidence)
F344_Male(low_confidence)
F344_Female(low_confidence)
F344_Both(low_confidence)
FHH_Male(low_confidence)
FHH_Female(low_confidence)
FHH_Both(confidence)
GH_Male(low_confidence)
GH_Female(low_confidence)
GH_Both(confidence)
LE_Male(low_confidence)
LE_Female(low_confidence)
LE_Both(low_confidence)
LEW_Male(low_confidence)
LEW_Female(low_confidence)
LEW_Both(low_confidence)
SHR_Male(low_confidence)
SHR_Female(low_confidence)
SHR_Both(low_confidence)
SS_Male(low_confidence)
SS_Female(low_confidence)
SS_Both(confidence)
WKY_Male(low_confidence)
WKY_Female(low_confidence)
WKY_Both(low_confidence)
0
100
200
300
serum aspartate aminotransferase activity level Meta-analysis Value

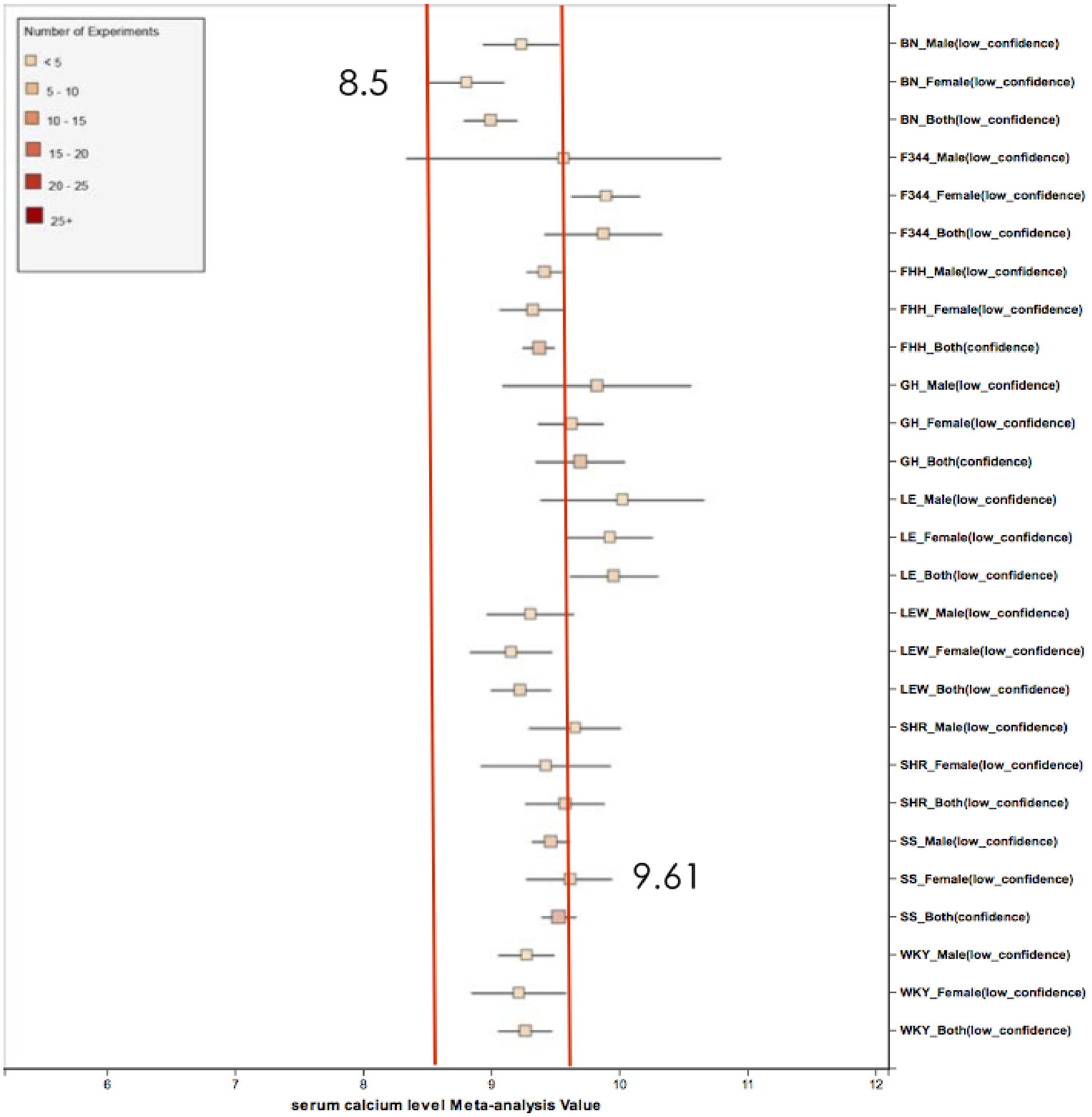
Number of Experiments
< 5
5 - 10
10 - 15
15 - 20
20 - 25
25+
8.5
9.61
BN_Male(low_confidence)
BN_Female(low_confidence)
BN_Both(low_confidence)
F344_Male(low_confidence)
F344_Female(low_confidence)
F344_Both(low_confidence)
FHH_Male(low_confidence)
FHH_Female(low_confidence)
FHH_Both(confidence)
GH_Male(low_confidence)
GH_Female(low_confidence)
GH_Both(confidence)
LE_Male(low_confidence)
LE_Female(low_confidence)
LE_Both(low_confidence)
LEW_Male(low_confidence)
LEW_Female(low_confidence)
LEW_Both(low_confidence)
SHR_Male(low_confidence)
SHR_Female(low_confidence)
SHR_Both(low_confidence)
SS_Male(low_confidence)
SS_Female(low_confidence)
SS_Both(confidence)
WKY_Male(low_confidence)
WKY_Female(low_confidence)
WKY_Both(low_confidence)
6
7
8
9
10
11
12
serum calcium level Meta-analysis Value

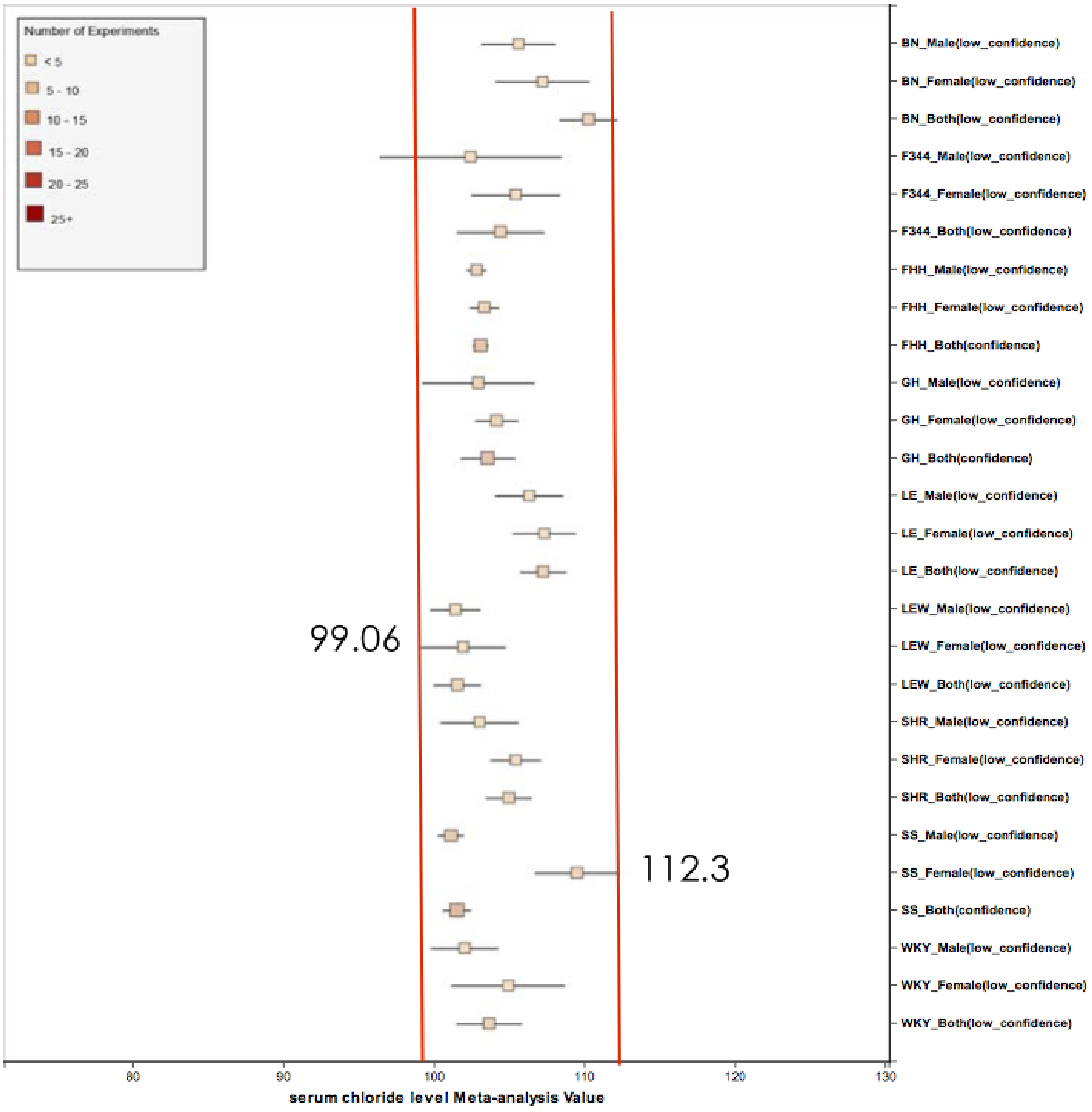
Number of Experiments
< 5
5 - 10
10 - 15
15 - 20
20 - 25
25+
99.06
112.3
BN_Male(low_confidence)
BN_Female(low_confidence)
BN_Both(low_confidence)
F344_Male(low_confidence)
F344_Female(low_confidence)
F344_Both(low_confidence)
FHH_Male(low_confidence)
FHH_Female(low_confidence)
FHH_Both(confidence)
GH_Male(low_confidence)
GH_Female(low_confidence)
GH_Both(confidence)
LE_Male(low_confidence)
LE_Female(low_confidence)
LE_Both(low_confidence)
LEW_Male(low_confidence)
LEW_Female(low_confidence)
LEW_Both(low_confidence)
SHR_Male(low_confidence)
SHR_Female(low_confidence)
SHR_Both(low_confidence)
SS_Male(low_confidence)
SS_Female(low_confidence)
SS_Both(confidence)
WKY_Male(low_confidence)
WKY_Female(low_confidence)
WKY_Both(low_confidence)
80
90
100
110
120
130
serum chloride level Meta-analysis Value

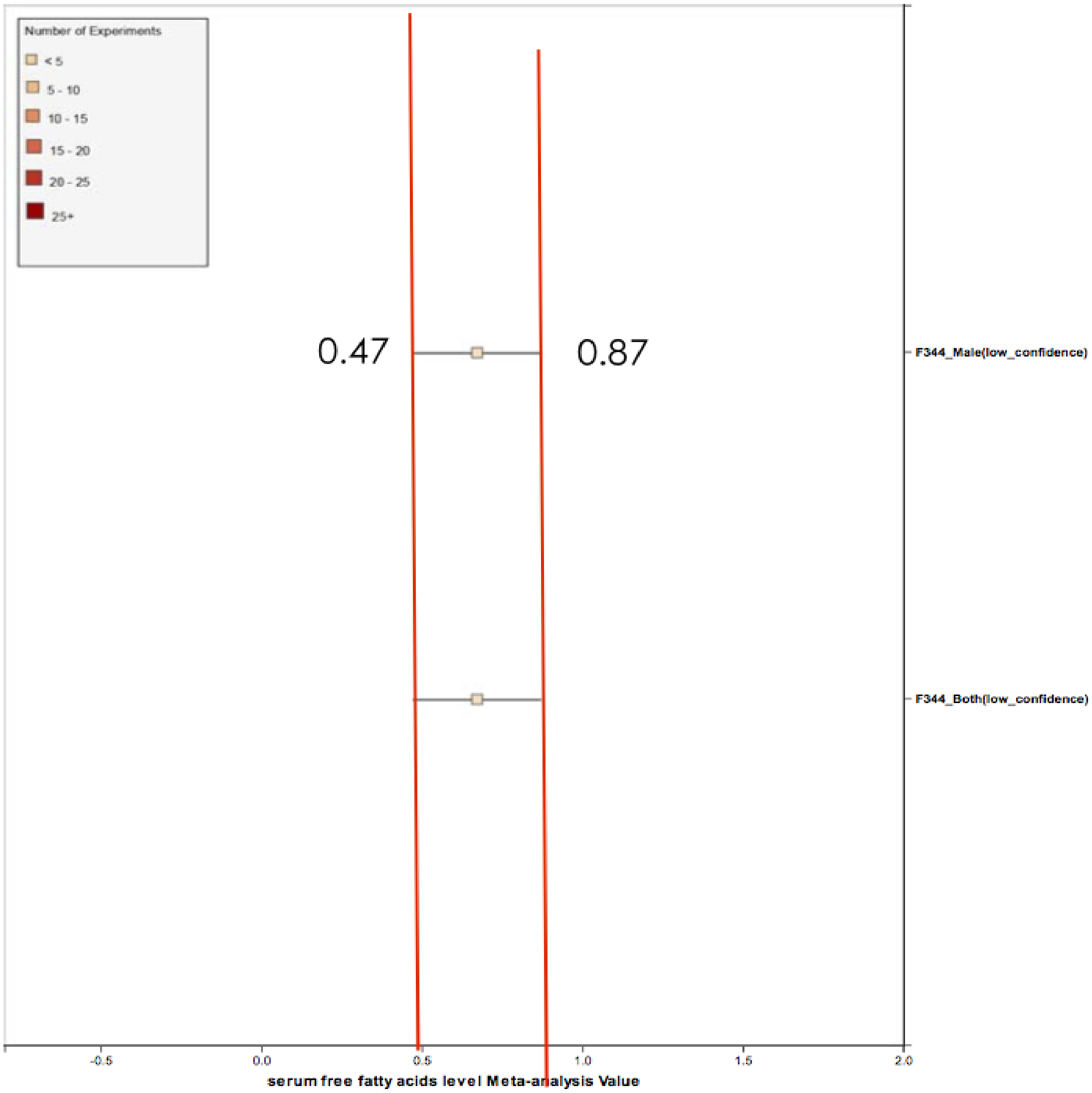
Number of Experiments
< 5
5 - 10
10 - 15
15 - 20
20 - 25
25+
0.47
0.87
F344_Male(low_confidence)
F344_Both(low_confidence)
-0.5
0.0
0.5
1.0
1.5
2.0
serum free fatty acids level Meta-analysis Value

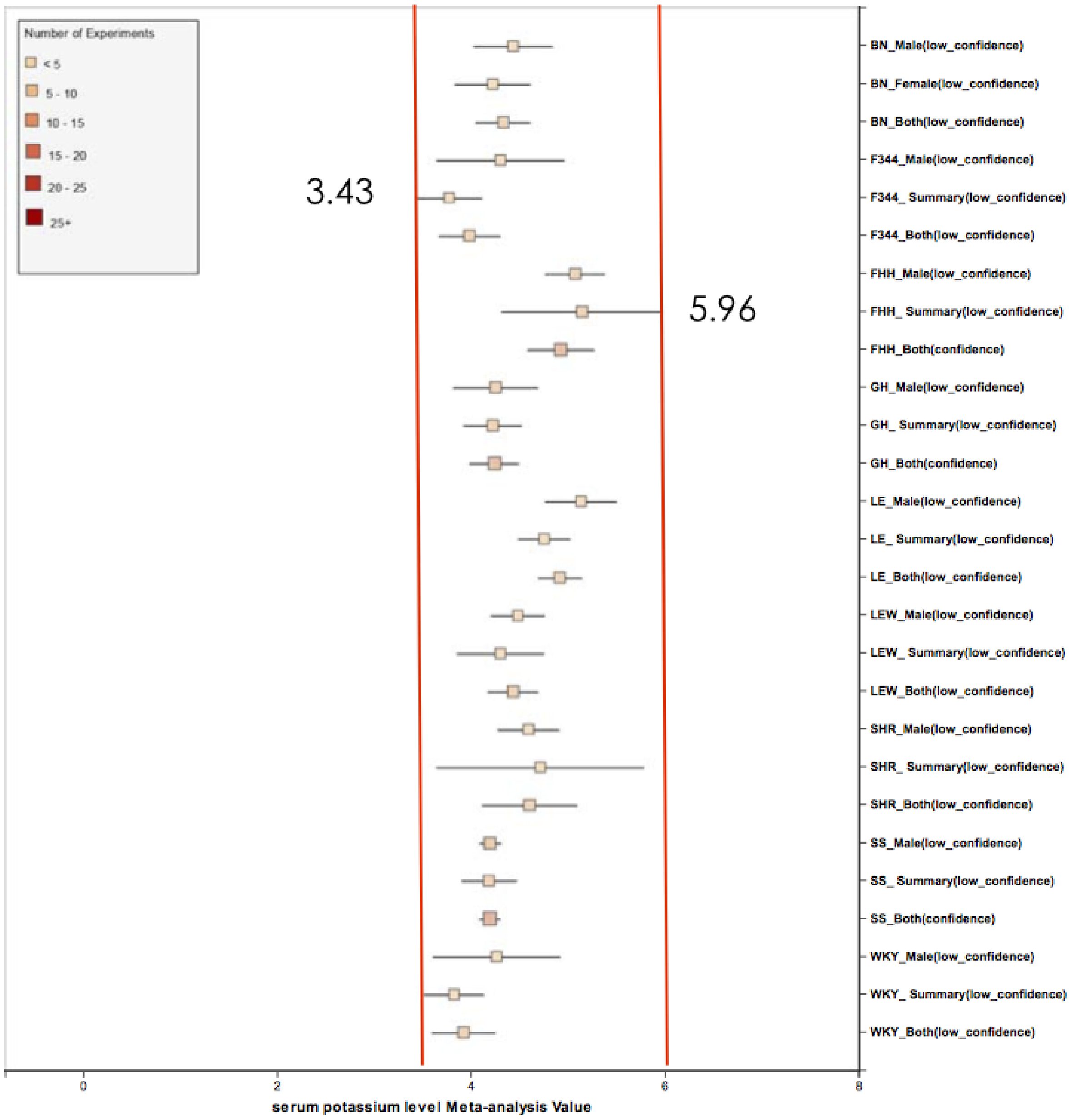

Number of Experiments
< 5
5 - 10
10 - 15
15 - 20
20 - 25
25+
3.43
5.96
BN_Male(low_confidence)
BN_Female(low_confidence)
BN_Both(low_confidence)
F344_Male(low_confidence)
F344_ Summary(low_confidence)
F344_Both(low_confidence)
FHH_Male(low_confidence)
FHH_ Summary(low_confidence)
FHH_Both(confidence)
GH_Male(low_confidence)
GH_ Summary(low_confidence)
GH_Both(confidence)
LE_Male(low_confidence)
LE_ Summary(low_confidence)
LE_Both(low_confidence)
LEW_Male(low_confidence)
LEW_ Summary(low_confidence)
LEW_Both(low_confidence)
SHR_Male(low_confidence)
SHR_ Summary(low_confidence)
SHR_Both(low_confidence)
SS_Male(low_confidence)
SS_ Summary(low_confidence)
SS_Both(confidence)
WKY_Male(low_confidence)
WKY_ Summary(low_confidence)
WKY_Both(low_confidence)
0
2
4
6
8
serum potassium level Meta-analysis Value

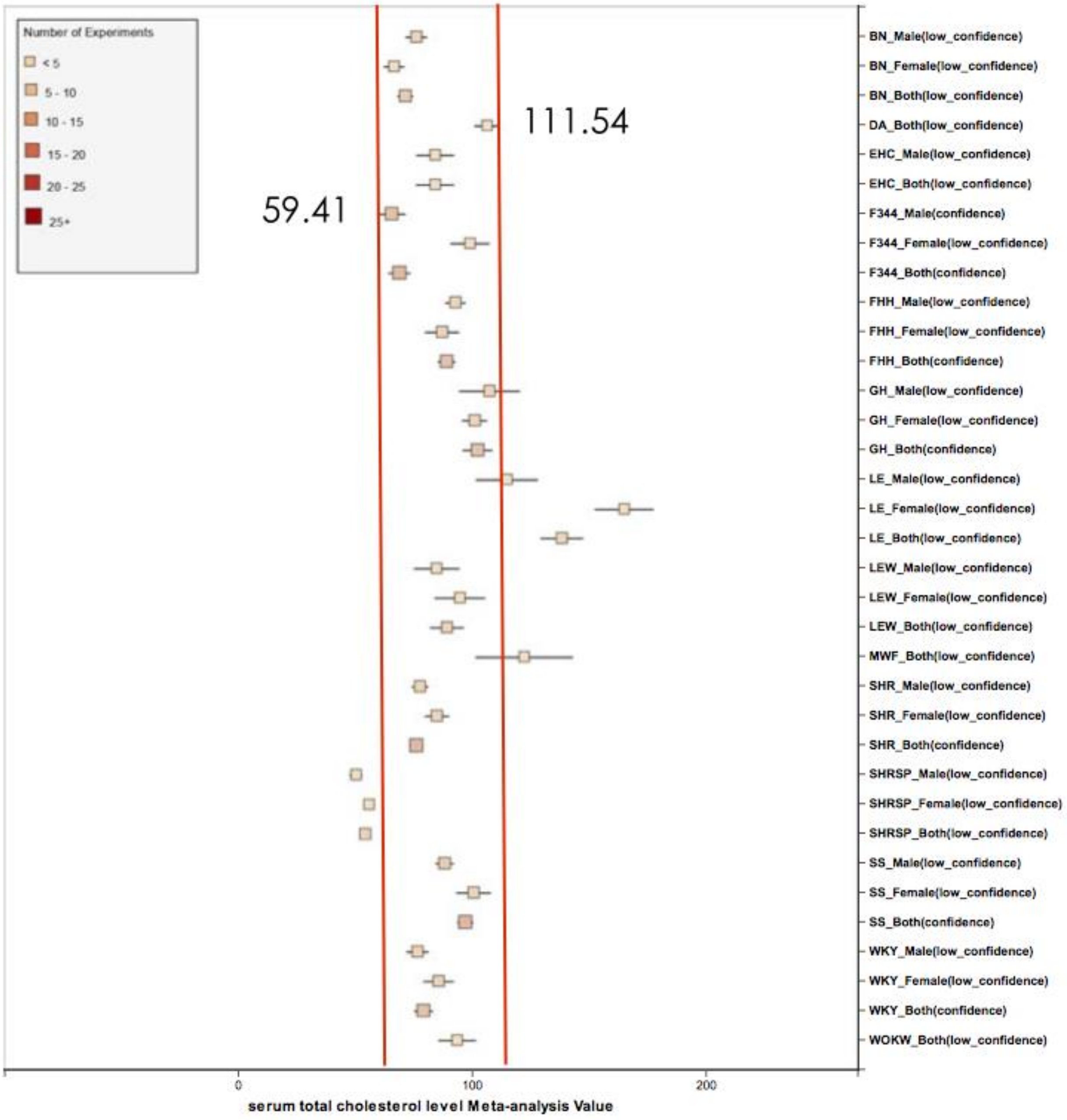

Number of Experiments
< 5
5 - 10
10 - 15
15 - 20
20 - 25
25+
111.54
59.41
BN_Male(low_confidence)
BN_Female(low_confidence)
BN_Both(low_confidence)
DA_Both(low_confidence)
EHC_Male(low_confidence)
EHC_Both(low_confidence)
F344_Male(confidence)
F344_Female(low_confidence)
F344_Both(confidence)
FHH_Male(low_confidence)
FHH_Female(low_confidence)
FHH_Both(confidence)
GH_Male(low_confidence)
GH_Female(low_confidence)
GH_Both(confidence)
LE_Male(low_confidence)
LE_Female(low_confidence)
LE_Both(low_confidence)
LEW_Male(low_confidence)
LEW_Female(low_confidence)
LEW_Both(low_confidence)
MWF_Both(low_confidence)
SHR_Male(low_confidence)
SHR_Female(low_confidence)
SHR_Both(confidence)
SHRSP_Male(low_confidence)
SHRSP_Female(low_confidence)
SHRSP_Both(low_confidence)
SS_Male(low_confidence)
SS_Female(low_confidence)
SS_Both(confidence)
WKY_Male(low_confidence)
WKY_Female(low_confidence)
WKY_Both(confidence)
WOKW_Both(low_confidence)
0
100
200
serum total cholesterol level Meta-analysis Value

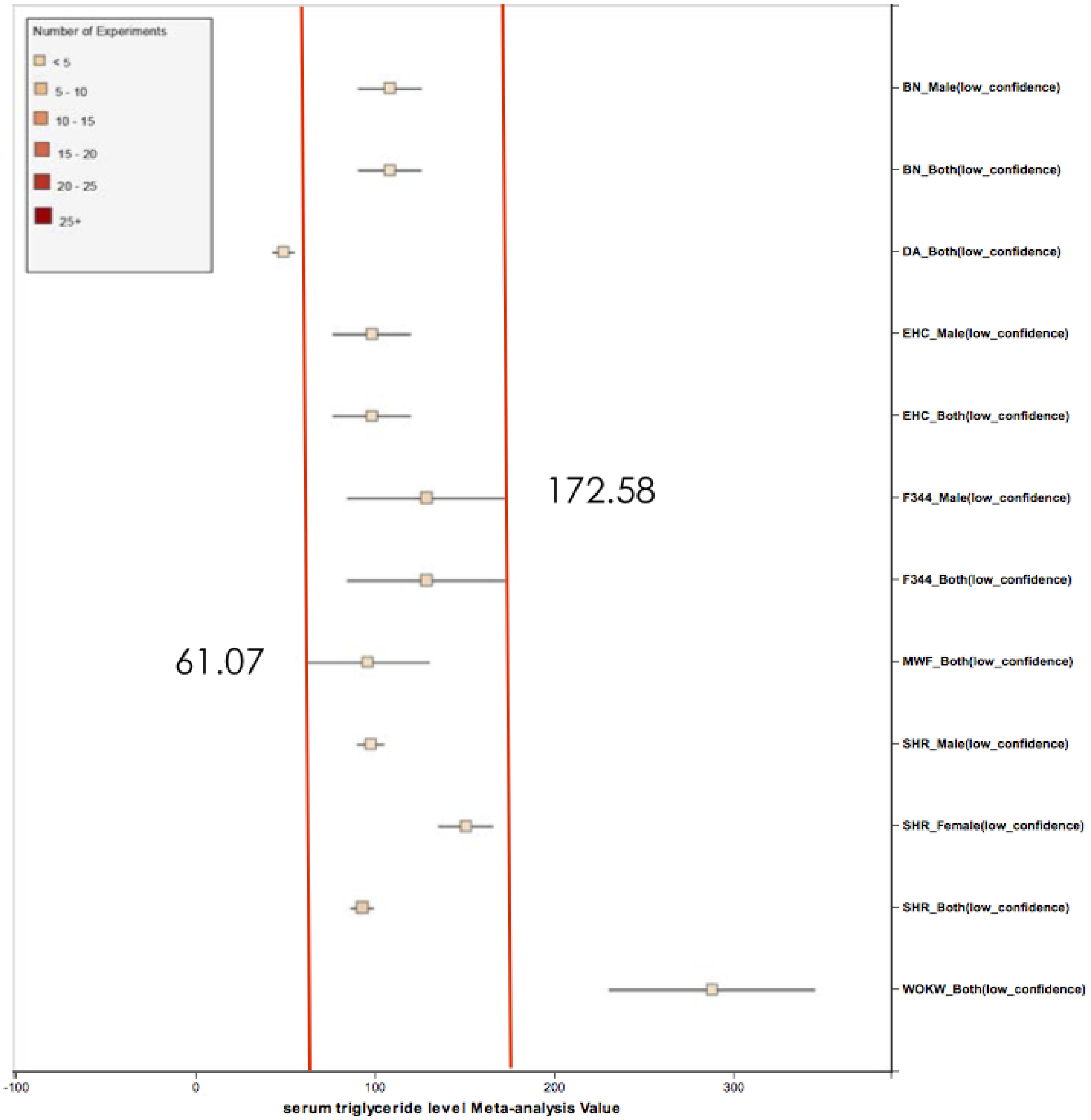
Number of Experiments
< 5
5 - 10
10 - 15
15 - 20
20 - 25
25+
172.58
61.07
BN_Male(low_confidence)
BN_Both(low_confidence)
DA_Both(low_confidence)
EHC_Male(low_confidence)
EHC_Both(low_confidence)
F344_Male(low_confidence)
F344_Both(low_confidence)
MWF_Both(low_confidence)
SHR_Male(low_confidence)
SHR_Female(low_confidence)
SHR_Both(low_confidence)
WOKW_Both(low_confidence)
-100
0
100
200
300
serum triglyceride level Meta-analysis Value

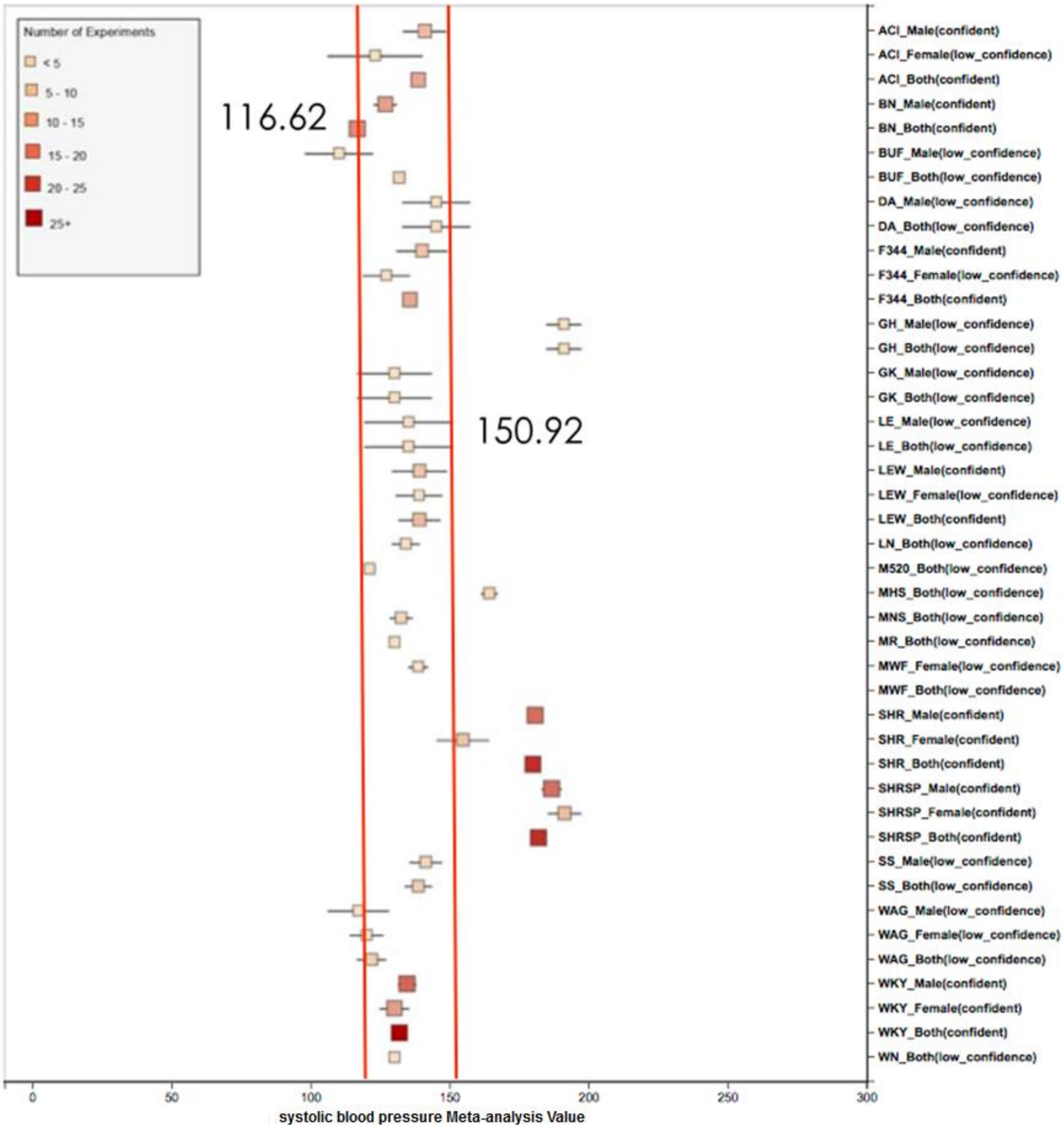
Number of Experiments
< 5
5 - 10
10 - 15
15 - 20
20 - 25
25+
116.62
150.92
ACI_Male(confident)
ACI_Female(low_confidence)
ACI_Both(confident)
BN_Male(confident)
BN_Both(confident)
BUF_Male(low_confidence)
BUF_Both(low_confidence)
DA_Male(low_confidence)
DA_Both(low_confidence)
F344_Male(confident)
F344_Female(low_confidence)
F344_Both(confident)
GH_Male(low_confidence)
GH_Both(low_confidence)
GK_Male(low_confidence)
GK_Both(low_confidence)
LE_Male(low_confidence)
LE_Both(low_confidence)
LEW_Male(confident)
LEW_Female(low_confidence)
LEW_Both(confident)
LN_Both(low_confidence)
M520_Both(low_confidence)
MHS_Both(low_confidence)
MNS_Both(low_confidence)
MR_Both(low_confidence)
MWF_Female(low_confidence)
MWF_Both(low_confidence)
SHR_Male(confident)
SHR_Female(confident)
SHR_Both(confident)
SHRSP_Male(confident)
SHRSP_Female(confident)
SHRSP_Both(confident)
SS_Male(low_confidence)
SS_Both(low_confidence)
WAG_Male(low_confidence)
WAG_Female(low_confidence)
WAG_Both(low_confidence)
WKY_Male(confident)
WKY_Female(confident)
WKY_Both(confident)
WN_Both(low_confidence)
0
50
100
150
200
250
300
systolic blood pressure Meta-analysis Value

SUPPLEMENTARY INFORMATION II: SUMMARY OF ABNORMAL AND NORMAL STRAINS FOR 24 CARDIOVASCULAR PHENOTYPES

Phenotype	Abnormal	Normal	Naïve Control
**blood hemoglobin level**		all	
**diastolic blood pressure**	GH, LH, SHR, SHRSP	BN, HTG, LEW, MHS, WKY	BN, WKY
**heart left ventricle weight to body weight ratio**	SHR	COP, DA, DRY, F344, LEW, WKY	WKY
**heart left ventricle wet weight**	GH	BN, COP, DA, DRY, F344, LEW, SHR, WKY	BN, WKY
**heart rate**	ACI, DA, GK	BN, BUF, F344, FHH, GH, LE, LEW, SHR, SHRSP, SS, WAG, WKY	BN, WKY
**heart right ventricle weight to left ventricle weight ratio**	F344, FHH, GH	LE, LEW, SHR, SS, WKY	WKY
**heart right ventricle wet weight**		F344, WKY	WKY
**heart weight as percentage of body weight**	FHH, SHR, SHRSP	ACI, BN, BUF, COP, DA, F344, GK, LE, LEW, SS, WAG, WKY	BN, WKY
**heart weight to body weight ratio**	SHRSP	ACI, BN, BUF, COP, DA, F344, ISIAH, M520, MWF, SHR, WAG, WKY, WN	BN, WKY
**heart wet weight**	BUF, FHH, GH, SS	ACI, BN, COP, DA, F344, LE, LEW, MWK, WAG, WKY	BN, WKY
**hematocrit**		all	
**mean arterial blood pressure**	GH, SHR, SHRSP	BN, LE, LEW, SS, WKY	BN, WKY
**mean corpuscular volume**		all	
**plasma total cholesterol level**		HTG, LEW	
**plasma triglyceride level**		COP, HTG, LEW	
**red blood cell count**		all	
**serum aspartate aminotransferase activity level**	SHR	BN, F344, FHH, GH, LE, LEW, SS, WKY	BN, WKY

**serum calcium level**	F344, GH, LE	BN, FHH, LEW, SHR, SS, WKY	BN, WKY
**serum chloride level**		all	
**serum free fatty acids level**		F344	
**serum potassium level**		all	
**serum total cholesterol level**	GH, MWF, SHRSP	BN, DA, EHC, F344, FHH, GH, LEW, SHR, SS, WKY, WOKW	BN, WKY
**serum triglyceride level**	DA, WOKW	BN, EHC, F344, MWF, SHR	BN
**systolic blood pressure**	GH, LH, MHS, SHR, SHRSP	ACI, BN, BUF, DA, F344, GK, LE, LEW, LN, M520, MNS, MR, MWF, WKY	BN, WKY

* ACI = August Copenhagen Irish, BN= Brown Norway, BUF= Buffalo, DA = Dark Agouti, F344 = Fischer 344, FHH = Fawn Hooded Hypertensive, GK = Goto Kakizaki, GH = Genetically Hypertensive, LH = Lyon Hypertensive, LEW = Lewis, SS = Dahl Salt-Sensitive, SHR = Spontaneously Hypertensive Rat, SHRSP = Spontaneously Hypertensive Rat, Stroke Prone, WKY = Wistar Kyoto

SUPPLEMENTARY INFORMATION III: AGREEMENTS BETWEEN META-ANALYSIS RESULTS AND DOMAIN EXPERT EMPIRICAL KNOWLEDGE

Phenotype	Agreement	Disagreement
blood hemoglobin level	31	0
diastolic blood pressure	23	0
heart left ventricle weight to body weight ratio	13	0
heart left ventricle wet weight	15	2
heart rate	41	1
heart right ventricle weight to left ventricle weight ratio	24	3
heart right ventricle wet weight	2	0
heart weight as percentage of body weight	36	3
heart weight to body weight ratio	20	0
heart wet weight	44	1
hematocrit	33	0
mean arterial blood pressure	30	0
mean corpuscular volume	31	0
plasma total cholesterol level	5	0
plasma triglyceride level	7	0
red blood cell count	27	0
serum aspartate aminotransferase activity level	27	0
serum calcium level	27	0
serum chloride level	27	0
serum free fatty acids level	2	0
serum potassium level	27	0
serum total cholesterol level	35	0
serum triglyceride level	12	0
systolic blood pressure	27	1
